# Gene-Based Methods for Estimating the Degree of the Skewness of X Chromosome Inactivation

**DOI:** 10.3390/genes13050827

**Published:** 2022-05-06

**Authors:** Meng-Kai Li, Yu-Xin Yuan, Bin Zhu, Kai-Wen Wang, Wing Kam Fung, Ji-Yuan Zhou

**Affiliations:** 1 Department of Biostatistics, State Key Laboratory of Organ Failure Research, Ministry of Education, and Guangdong Provincial Key Laboratory of Tropical Disease Research, School of Public Health, Southern Medical University, Guangzhou 510515, China; lmksum@163.com (M.-K.L.); yuxinyuan2000@163.com (Y.-X.Y.); labenjay@163.com (B.Z.); wkw18927270041@hotmail.com (K.-W.W.); 2 Guangdong-Hong Hong-Macao Joint Laboratory for Contaminants Exposure and Health, Guangzhou 510006, China; 3 Department of Statistics and Actuarial Science, The University of Hong Kong, Hong Kong, China; wingfung@hku.hk

**Keywords:** skewed X chromosome inactivation, Fieller’s method, penalized Fieller’s method, Bayesian method, Minnesota Center for Twin and Family Research data

## Abstract

Skewed X chromosome inactivation (XCI-S) has been reported to be associated with some X-linked diseases, and currently several methods have been proposed to estimate the degree of the XCI-S (denoted as γ) for a single locus. However, no method has been available to estimate γ for genes. Therefore, in this paper, we first propose the point estimate and the penalized point estimate of γ for genes, and then derive its confidence intervals based on the Fieller’s and penalized Fieller’s methods, respectively. Further, we consider the constraint condition of γ∈[0, 2] and propose the Bayesian methods to obtain the point estimates and the credible intervals of γ, where a truncated normal prior and a uniform prior are respectively used (denoted as GBN and GBU). The simulation results show that the Bayesian methods can avoid the extreme point estimates (0 or 2), the empty sets, the noninformative intervals ([0, 2]) and the discontinuous intervals to occur. GBN performs best in both the point estimation and the interval estimation. Finally, we apply the proposed methods to the Minnesota Center for Twin and Family Research data for their practical use. In summary, in practical applications, we recommend using GBN to estimate γ of genes.

## 1. Introduction

X chromosome inactivation (XCI) is an important epigenetic phenomenon. Under the XCI, one of two X chromosomes in females is silenced in the early stage of embryonic development to ensure that the transcriptional dosage of X chromosomes in females and that in males are balanced [1]. Generally, there are three patterns of the XCI [2], random X chromosome inactivation (XCI-R), skewed X chromosome inactivation (XCI-S) [3,4,5,6], and escape from X chromosome inactivation (XCI-E) [7,8]. The XCI-R means that the paternal and maternal X chromosomes in females have the same probabilities to be inactive, i.e., for a locus on the X chromosome, approximately 50% of the cells inactivate one of the alleles, while the remaining 50% of the cells keep the other allele inactive. Under the XCI-E, the alleles on both the X chromosomes in females are expressed, which are similar to those at an autosomal locus. For humans, about 15-30% of the X-linked genes have been reported to undergo the XCI-E [7]. Finally, the XCI-S is defined as more than 75% of the cells in females inactivating the same allele [9]. For some extreme skewed cases, it is possible that more than 90% of the cells keep the same allele silenced [9,10]. As such, the difference in the number of the X chromosomes in females and males and the complexity of the XCI make the association tests for the X chromosomes more complicated than those for the autosomes.

The skewness of the XCI can reflect, or cause, biological consequences for females [9]. The clonal expansion of a somatic cell in females may lead to a cell population with extremely skewed XCI [9]. For some X-linked disorders, there is strong selection of the cells which keep the mutant allele inactive in the heterozygous carriers and, hence, assessing the degree of the skewness of the XCI is helpful in terms of being indicative of the carrier’s disease status [11]. Further, the degree of the skewness of the XCI can determine the severity of certain X-linked diseases, such as haemophilia B [12,13]. On the other hand, even for the same mutant allele, the XCI-S in different tissues or cells may result in different clinical consequences. For example, in heterozygous females with a mutant FoxP3 allele, the XCI-S against the mutant allele in specific tissues can prevent autoimmune disease, while the XCI-S skewed towards the mutant allele in breast epithelial cells can cause breast cancer [14]. Besides this, studies have shown that some diseases, such as ovarian cancer, Rett syndrome, Duchenne muscular dystrophy and recurrent miscarriage, are also related to the XCI-S [15,16,17,18]. Therefore, in recent years, researchers have proposed some methods to test the association between the alleles at an X-chromosomal single nucleotide polymorphism (SNP) locus and traits [19,20,21,22,23,24,25,26]. For example, Wang et al. [23] developed a permutation-based test statistic which considers all the XCI patterns. For the XCI-R and the XCI-S, this method respectively codes three female genotypes (dd, Dd and DD) as 0, γ and 2 at an X-chromosomal SNP, with the major allele d and the minor allele D, where γ∈[0, 2] is an unknown genotypic value for heterozygous females, and respectively codes two male genotypes (d and D) as 0 and 2. Here, γ can be used to measure the degree of the XCI skewing. For instance, γ∈[0, 1) is indicative of the XCI-S skewed towards the minor allele D, γ=1 means that the XCI pattern is the XCI-R, and γ∈(1, 2] indicates the XCI-S skewed towards the major allele d. For the XCI-E, three female genotypes are coded as 0, 1 and 2, and two male genotypes are coded as 0 and 1, respectively. However, the X-chromosomal association tests mentioned above are only applicable to a single SNP and common variants, and are not suitable for genetic regions or genes with multiple SNPs and rare variants. Rare variants refer to the variants with a minor allele frequency (MAF) less than 1%, and those with MAF ≥1% are called common variants [27,28]. Over the past few years, genome-wide association studies have identified many common variants associated with complex traits, but these variants usually explain only a small part of the estimated heritability for a given trait. On the other hand, it has been shown that rare variants play a key role in influencing traits [29]. Single-variant tests often have low test power when applied to the rare variants. Therefore, many statistical methods had been presented, which focus on testing the cumulative effect of rare variants in genetic regions or SNP sets (such as genes), including the burden test and the variance-component tests [27,30,31,32,33]. The burden test collapses all the rare variants in a genetic region into a single burden variable, and then regresses the trait on the burden variable to test the cumulative effect of the rare variants in that region [27]. The variance-component tests, such as the sequence kernel association test (SKAT), do not directly aggregate the variants in the modeling process, but aggregate the association between the variants and the trait through a kernel matrix [33]. Another method, SKAT-O, proposed by Lee et al. [34], has the advantages of both the burden and SKAT tests, but the time cost is higher than the previous two methods. All these methods have one thing in common, i.e., increasing the weights of rare variants’ contributions and decreasing the weights of common variants’ contributions. However, for a trait-related gene, the relative influence of rare and common variants is not known [35]. Therefore, Iuliana et al. [35] put forward several multi-locus association tests, such as the adaptive sum test, which consider the effects of both common and rare variants on the trait, and these methods are more powerful when the genes simultaneously contain rare and common variants. Note that these multi-locus association tests are all based on genetic regions or genes on autosomes, and may not be directly applied to the X chromosomes. Therefore, Clement et al. [36] improved the traditional burden test, SKAT and SKAT-O methods and suggested three gene-based X-chromosomal association tests. However, these methods only take account of the XCI-R and XCI-E patterns. What is more, the FxSKAT method, proposed by Asuman et al. [37], is not only applicable to pedigree data, but also takes the XCI-E into account during the analysis process.

Except for testing the association between the genes on the X chromosome and the traits under study, it is also important to develop methods to measure the corresponding degree of the skewness of the XCI (denoted as γ). At present, researchers have put forward several methods to estimate γ for a single SNP, which can simultaneously get the point estimates and the confidence intervals (CIs) of γ. Specifically, Xu et al. [38] proposed a statistical index for estimating γ based on family trios (both parents and their daughter), which can be represented as the ratio of two relative risks in females, and derived the corresponding CI with the likelihood ratio (LR) test. Wang et al. [39] used the ratio of two regression coefficients of a logistic regression to estimate γ, and obtained the CIs with the LR, Fieller’s and delta methods, respectively. Li et al. [40] further extended the methods of Wang et al. so that they can accommodate quantitative traits. However, the above-mentioned methods are all constructed for a single SNP, and are not suitable for genetic regions or genes containing multiple SNPs. Furthermore, when applied to rare variants, they perform poorly. In addition, it should be noted that the delta method cannot control the coverage probability (CP) well, and the LR and Fieller’s methods have similar performance in the interval estimation, while the Fieller’s method is computationally efficient. Thus, the Fieller’s method is recommended in practice. However, both the LR and Fieller’s methods may yield unbounded CIs when the denominators in the ratios used to estimate γ are close to 0. Fortunately, the penalized Fieller’s (PF) method, which was proposed by Wang et al. [41], can be used to conduct the ratio estimation and always get the bounded CIs by choosing an appropriate penalty parameter. However, it has not been applied to the estimation of the degree of the skewness of the XCI yet. On the other hand, the above-mentioned methods do not consider the constraint condition of γ∈[0, 2], and simply cut off the point estimates and the CIs within [0, 2], which may result in extreme point estimates (0 or 2) and empty sets or noninformative CIs (i.e., [0, 2]). In contrast, the Bayesian methods can effectively utilize the prior information of each unknown parameter in the analysis, and have been widely used in statistical genetics [42].

Therefore, in this paper, we borrow the idea of the burden test, aggregate all the variants in a gene under study into a burden variable by selecting the appropriate weights, and then estimate the mean degree of the skewness of the XCI over all the SNPs in the gene based on the burden variable. We first propose the point estimate and the penalized point estimate of γ for the gene, and then derive its CIs based on the Fieller’s and PF methods, respectively. Then, by considering the constraint condition of γ∈[0, 2], we propose the Bayesian methods to obtain the point estimates and the credible intervals of γ. Specifically, after getting enough samples drawn from the posterior distribution of γ, we calculate the mode of the samples as the point estimate of γ and the highest posterior density interval (HPDI) as the credible interval of γ [43]. We conduct extensive simulation studies to compare the performances of the proposed point estimation methods and the interval estimation methods for γ. Finally, we demonstrate the practical utility of the proposed methods by applying them to the Minnesota Center for Twin and Family Research (MCTFR) data.

## 2. Materials and Methods

### 2.1. Notations

Suppose that we only collect n female subjects, because male subjects provide no information on the XCI skewing. Consider an X-linked trait (quantitative or qualitative) and let $y_{i}$ represent the trait value of the ith female (i=1, 2, …, n), then $Y={\left(y_{1},y_{2},\;\ldots ,y_{n}\right)}^{T}$ is the vector of the trait values for all the females. Assume that a gene which contains J SNPs is associated with this trait, and let $d_{j}$ and $D_{j}$ denote the major allele and the minor allele at the jth SNP (j=1, 2, …, J), respectively. Let $G_{ij}$ be the genotype at the jth SNP of the ith female (i.e., $G_{ij}=d_{j}d_{j},\;D_{j}d_{j}$ or $D_{j}D_{j}$). If we use γ∈[0, 2] to measure the mean degree of the skewness of the XCI for all the SNPs in the gene, then $g_{ij}=0,\;\gamma$ and 2 can be used to denote the genotypic values for genotypes $d_{j}d_{j}$, $D_{j}d_{j}$ and $D_{j}D_{j}$, respectively. As such, $G_{i}={\left(g_{i1},g_{i2},\ldots ,g_{iJ}\right)}^{T}$ is the vector of the genotypic values at the J SNPs of the ith female. Therefore, we consider the association between the gene and the trait based on the following generalized linear model

(1) $$h\left(\mu_{i}\right)=\beta_{0}+\beta^{T}G_{i}+b^{T}Z_{i},$$

where  h(·) is a link function; $Z_{i}={\left(Z_{i1},Z_{i2},\ldots ,Z_{im}\right)}^{T}$ is the vector of m covariates of the ith female, which are needed to be adjusted, and $Z={\left(Z_{1},\;Z_{2},\;\ldots ,Z_{n}\right)}^{T}$ is an n×m covariate matrix; $\mu_{i}=E\left(y_{i}|G_{i},Z_{i}\right)$ is the conditional mean of the ith female’s trait value given $G_{i}$ and $Z_{i}$; $\beta_{0}$ is the intercept, $\beta ={\left(\beta_{1},\beta_{2},\ldots ,\beta_{J}\right)}^{T}$ is the vector of the regression coefficients of $G_{i}$, and $b={\left(b_{1},b_{2},\ldots ,b_{m}\right)}^{T}$ is an m×1 vector of the regression coefficients of $Z_{i}$.

Based on the idea of the burden test [27], we aggregate all the SNPs in the gene into a burden variable and let $X_{i}=\overset{J}{\underset{j=1}{\sum }}\omega_{j}g_{ij}$, where $\omega_{j}$ is a weight for the jth SNP. Here we assume that $\omega_{j}$ is a function with respect to the MAF at the jth SNP (denoted as ${\mathrm{MAF}}_{j}$), i.e., $\omega_{j}=Beta\left({\mathrm{MAF}}_{j},0.5,\;0.5\right)$ [35]. So, model (1) can be rewritten as

(2) $$h\left(\mu_{i}\right)=\beta_{0}+\beta_{c}X_{i}+b^{T}Z_{i},$$

where $\beta_{c}$ is the regression coefficient of $X_{i}$. Next, we consider two variables $g_{ij}^{\left(1\right)}=I_{\left\{G_{ij}=D_{j}d_{j}\;\mathrm{or}\;D_{j}D_{j}\right\}}$ and $g_{ij}^{\left(2\right)}=I_{\left\{G_{ij}=D_{j}D_{j}\right\}}$, where $I_{\left\{\cdot \right\}}$ is the indicator function. Thus, $g_{ij}^{\left(1\right)}=1$ means that the ith female contains at least one minor allele at the jth SNP, and $g_{ij}^{\left(2\right)}=1$ denotes that the female is a homozygote $D_{j}D_{j}$ at the jth SNP. Through simple transformations, we can get $g_{ij}=\gamma g_{ij}^{\left(1\right)}+\left(2-\gamma \right)g_{ij}^{\left(2\right)}$, and $X_{i}$ can be expressed as $X_{i}=\overset{J}{\underset{j=1}{\sum }}\omega_{j}\left[\gamma g_{ij}^{\left(1\right)}+\left(2-\gamma \right)g_{ij}^{\left(2\right)}\right]=\gamma X_{i}^{\left(1\right)}+\left(2-\gamma \right)X_{i}^{\left(2\right)}$, where $X_{i}^{\left(1\right)}=\overset{J}{\underset{j=1}{\sum }}\omega_{j}g_{ij}^{\left(1\right)}$ and $X_{i}^{\left(2\right)}=\overset{J}{\underset{j=1}{\sum }}\omega_{j}g_{ij}^{\left(2\right)}$. Further, let $X^{\left(1\right)}={\left(X_{1}^{\left(1\right)},X_{2}^{\left(1\right)},\;\ldots ,X_{n}^{\left(1\right)}\right)}^{T}$ and $X^{\left(2\right)}={\left(X_{1}^{\left(2\right)},X_{2}^{\left(2\right)},\;\ldots ,X_{n}^{\left(2\right)}\right)}^{T}$. To estimate the mean degree of the XCI skewing for the gene (i.e., γ), we substitute $X_{i}=\gamma X_{i}^{\left(1\right)}+\left(2-\gamma \right)X_{i}^{\left(2\right)}$ into model (2) and get

(3) $$h\left(\mu_{i}\right)=\beta_{0}+\beta_{c}\left[\gamma X_{i}^{\left(1\right)}+\left(2-\gamma \right)X_{i}^{\left(2\right)}\right]+b^{T}Z_{i}.$$

For quantitative traits, h(·) is the identity function, and model (3) can be written as $y_{i}=\beta_{0}+\beta_{c}\left[\gamma X_{i}^{\left(1\right)}+\left(2-\gamma \right)X_{i}^{\left(2\right)}\right]+b^{T}Z_{i}+\varepsilon_{i}$, where $\varepsilon_{i}$ is the random error and follows $N\left(0,\;\sigma^{2}\right)$. In this case, the unknown parameters are $\theta_{1}={\left(\beta_{0},\beta_{c},\gamma ,b^{T},\sigma \right)}^{T}$, and the corresponding likelihood function of the sample is

$$L_{1}\left(\theta_{1}\right)={\left(2\pi \sigma^{2}\right)}^{\sum \frac{n}{2}}\exp\left\{-\frac{\sum_{i=1}^{n}{\left[y_{i}-\beta_{0}-\gamma \beta_{c}X_{i}^{\left(1\right)}-\left(2-\gamma \right)\beta_{c}X_{i}^{\left(2\right)}-b^{T}Z_{i}\right]}^{2}}{2\sigma^{2}}\right\}.$$

As for qualitative traits, h(·) is the logit function, and model (3) is written as $\mathrm{Logit}\left(Pr\left(y_{i}=1|X_{i}^{\left(1\right)},X_{i}^{\left(2\right)},Z_{i}\right)\right)$=$\beta_{0}+\beta_{c}\left[\gamma X_{i}^{\left(1\right)}+\left(2-\gamma \right)X_{i}^{\left(2\right)}\right]+b^{T}Z_{i}$. The unknown parameters are $\theta_{2}={\left(\beta_{0},\beta_{c},\gamma ,b^{T}\right)}^{T}$ and the likelihood function is

$$L_{2}\left(\theta_{2}\right)=\prod_{i=1}^{n}\pi_{i}{}^{I_{\left\{y_{i}=1\right\}}}{\left(1-\pi_{i}\right)}^{I_{\left\{y_{i}=0\right\}}},$$

where $y_{i}=1$ and 0 respectively indicate that the ith female is a case and a control, and $\pi_{i}=1/\left\{1+\exp\left[-\beta_{0}-\gamma \beta_{c}X_{i}^{\left(1\right)}-\left(2-\gamma \right)\beta_{c}X_{i}^{\left(2\right)}-b^{T}Z_{i}\right]\right\}$. Let $\beta_{c}^{\left(1\right)}=\gamma \beta_{c}$ and $\beta_{c}^{\left(2\right)}=\left(2-\gamma \right)\beta_{c}$, and we have

(4) $$h\left(\mu_{i}\right)=\beta_{0}+\beta_{c}^{\left(1\right)}X_{i}^{\left(1\right)}+\beta_{c}^{\left(2\right)}X_{i}^{\left(2\right)}+b^{T}Z_{i}.$$

As such, we obtain $\beta_{c}=\left(\beta_{c}^{\left(1\right)}+\beta_{c}^{\left(2\right)}\right)/2$ and γ can be expressed as

(5) $$\gamma =\frac{\beta_{c}^{\left(1\right)}}{\beta_{c}}=\frac{2\beta_{c}^{\left(1\right)}}{\beta_{c}^{\left(1\right)}+\beta_{c}^{\left(2\right)}}.$$

By assuming that the degree of the skewness of the XCI at the jth SNP is $\gamma_{j}$, γ satisfies, under a certain condition (the proof is given in Appendix A),

$$\gamma =\frac{\sum_{j=1}^{J}\omega_{j}\left(g_{.j}^{\left(1\right)}-g_{.j}^{\left(2\right)}\right)\gamma_{j}}{\sum_{j=1}^{J}\omega_{j}\left(g_{.j}^{\left(1\right)}-g_{.j}^{\left(2\right)}\right)},$$

where $g_{.j}^{\left(1\right)}=\overset{n}{\underset{i=1}{\sum }}g_{ij}^{\left(1\right)}$ is the number of the females who contain at least one minor allele at the jth SNP, and $g_{.j}^{\left(2\right)}=\overset{n}{\underset{i=1}{\sum }}g_{ij}^{\left(2\right)}$ is the number of the females whose genotypes at the jth SNP are $D_{j}D_{j}$. So, γ is the weighted mean of the $\gamma_{j}$’s for all the SNPs in the gene with the weights being $\omega_{j}\left(g_{.j}^{\left(1\right)}-g_{.j}^{\left(2\right)}\right)/\overset{J}{\underset{j=1}{\sum }}\omega_{j}\left(g_{.j}^{\left(1\right)}-g_{.j}^{\left(2\right)}\right)$. When there are rare variants at some SNPs or when the variation of the $\gamma_{j}$’s in the gene is large, γ is still well defined for the whole gene. On the other hand, from Equation (5), γ can be well defined if there is an association between the gene and the trait (i.e., $\beta_{c}=\left(\beta_{c}^{\left(1\right)}+\beta_{c}^{\left(2\right)}\right)/2\neq 0$). Further,  γ=0 if and only if $\beta_{c}^{\left(1\right)}=0$ and $\beta_{c}^{\left(2\right)}\neq 0$, which means that all the $\gamma_{j}$’s are 0 and the XCI-S is completely skewed towards the minor allele for each SNP, and γ=2 only when $\beta_{c}^{\left(1\right)}\neq 0$ and $\beta_{c}^{\left(2\right)}=0$, indicating that all the $\gamma_{j}$’s are 2 and the XCI-S is completely skewed towards the major allele for each SNP. However, γ=1 means that on the average, the gene undergoes the XCI-R or the XCI-E. After obtaining the estimates of $\beta_{c}^{\left(1\right)}$ and $\beta_{c}^{\left(2\right)}$, respectively denoted by ${\hat{\beta }}_{c}^{\left(1\right)}$ and ${\hat{\beta }}_{c}^{\left(2\right)}$ which can be derived by the maximum likelihood method, the point estimate of γ can be expressed as $\hat{\gamma }=2{\hat{\beta }}_{c}^{\left(1\right)}/\left({\hat{\beta }}_{c}^{\left(1\right)}+{\hat{\beta }}_{c}^{\left(2\right)}\right)$.

### 2.2. Point Estimate and CI of γ by Fieller’s Method

Note that γ should take the possible values from the interval [0, 2]. So, the original estimate $\hat{\gamma }=2{\hat{\beta }}_{c}^{\left(1\right)}/\left({\hat{\beta }}_{c}^{\left(1\right)}+{\hat{\beta }}_{c}^{\left(2\right)}\right)$ needs to be cut off in [0, 2] and the resulting estimate is denoted by ${\hat{\gamma }}_{GF}$. Further, we utilize the Fieller’s method to get the CI of γ. Specifically, borrowing the idea of Wang et al. [39], we have ${\hat{\beta }}_{c}=\left({\hat{\beta }}_{c}^{\left(1\right)}+{\hat{\beta }}_{c}^{\left(2\right)}\right)/2$, $\hat{\mathrm{Var}}\left({\hat{\beta }}_{c}\right)=\frac{1}{4}\left[\hat{\mathrm{Var}}\left({\hat{\beta }}_{c}^{\left(1\right)}\right)+\hat{\mathrm{Var}}\left({\hat{\beta }}_{c}^{\left(2\right)}\right)+2\hat{\mathrm{Cov}}\left({\hat{\beta }}_{c}^{\left(1\right)},{\hat{\beta }}_{c}^{\left(2\right)}\right)\right]$ and $\hat{\mathrm{Cov}}\left({\hat{\beta }}_{c}^{\left(1\right)},{\hat{\beta }}_{c}\right)=\frac{1}{2}\hat{\mathrm{Var}}\left({\hat{\beta }}_{c}^{\left(1\right)}\right)+\frac{1}{2}\hat{\mathrm{Cov}}\left({\hat{\beta }}_{c}^{\left(1\right)},{\hat{\beta }}_{c}^{\left(2\right)}\right)$. To construct the CI of γ, we first establish a Wald test under the null hypothesis $H_{0}:\;\gamma =\gamma_{0}$, where $\gamma_{0}$ is a pre-specified value (e.g., 1, which means that on the average, the gene undergoes the XCI-R or the XCI-E). As such, we have $\beta_{c}^{\left(1\right)}-\gamma_{0}\beta_{c}=0$, and the Wald test statistic is

$$\frac{{\hat{\beta }}_{c}^{\left(1\right)}-\gamma_{0}{\hat{\beta }}_{c}}{\sqrt{\hat{\mathrm{Var}}\left({\hat{\beta }}_{c}^{\left(1\right)}\right)+\gamma_{0}^{2}\hat{\mathrm{Var}}\left({\hat{\beta }}_{c}\right)-2\gamma_{0}\hat{\mathrm{Cov}}\left({\hat{\beta }}_{c}^{\left(1\right)},\;{\hat{\beta }}_{c}\right)}}\sim N\left(0,\;1\right).$$

Therefore, the 100(1−α)% CI of γ can be derived by solving the following equation

$${\left[\frac{{\hat{\beta }}_{c}^{\left(1\right)}-\gamma_{0}{\hat{\beta }}_{c}}{\sqrt{\hat{\mathrm{Var}}\left({\hat{\beta }}_{c}^{\left(1\right)}\right)+\gamma_{0}^{2}\hat{\mathrm{Var}}\left({\hat{\beta }}_{c}\right)-2\gamma_{0}\hat{\mathrm{Cov}}\left({\hat{\beta }}_{c}^{\left(1\right)},\;{\hat{\beta }}_{c}\right)}}\right]}^{2}=Z_{1-\alpha /2}^{2},$$

where $Z_{1-\alpha /2}$ is the (1−α/2) upper quantile of the standard normal distribution. Rearrange the above equation with respect to $\gamma_{0}$ into a quadratic equation

(6) $$A\gamma_{0}^{2}+B\gamma_{0}+C=0,$$

where $A={\hat{\beta }}_{c}^{2}-Z_{1-\alpha /2}^{2}\hat{\mathrm{Var}}\left({\hat{\beta }}_{c}\right)$, $B=2\left[Z_{1-\alpha /2}^{2}\hat{\mathrm{Cov}}\left({\hat{\beta }}_{c}^{\left(1\right)},{\hat{\beta }}_{c}\right)-{\hat{\beta }}_{c}^{\left(1\right)}{\hat{\beta }}_{c}\right]$ and $C={\left({\hat{\beta }}_{c}^{\left(1\right)}\right)}^{2}-Z_{1-\alpha /2}^{2}\hat{\mathrm{Var}}\left({\hat{\beta }}_{c}^{\left(1\right)}\right)$. When $\Delta =\sqrt{B^{2}-4AC}=0$ or A=0, the CI of γ will degenerate to be a point. The CI of γ for other cases is as follows

$$\{\begin{array}{r} \left(\frac{-B-\sqrt{\Delta }}{2A},\;\frac{-B+\sqrt{\Delta }}{2A}\right)\cap \left[0,\;2\right],\;\mathrm{if}\;\Delta >0\;\mathrm{and}\;A>0\\ \left(\left(-\infty ,\;\frac{-B+\sqrt{\Delta }}{2A}\right)\cup \left(\frac{-B-\sqrt{\Delta }}{2A},+\infty \right)\right)\cap \left[0,\;2\right],\;\mathrm{if}\;\Delta >0\;\mathrm{and}\;A<0\\ \left[0,\;2\right],\;\mathrm{if}\;\Delta <0\;\mathrm{and}\;A<0\\ \emptyset ,\;\mathrm{if}\;\Delta <0\;\mathrm{and}\;A>0 \end{array}$$

It should be noted that even in the case of Δ>0, the CI of γ obtained by the Fieller’s method may still be an empty set. And in the case of Δ>0 and A<0, the CI may be composed of two parts, which is the discontinuous interval.

### 2.3. Penalized Point Estimate and CI of γ by PF Method

As mentioned above, we construct $\hat{\gamma }={\hat{\beta }}_{c}^{\left(1\right)}/{\hat{\beta }}_{c}$ as the point estimate of γ, where ${\hat{\beta }}_{c}=\left({\hat{\beta }}_{c}^{\left(1\right)}+{\hat{\beta }}_{c}^{\left(2\right)}\right)/2$. However, if the denominator ${\hat{\beta }}_{c}$ is very close to 0, $\hat{\gamma }$ will tend to the infinity. The CI of γ based on the Fieller’s method before the truncation is usually unbounded. To deal with this issue in the ratio estimate and borrow the idea of Wang et al. [41], we propose the following PF method to obtain the penalized point estimate of γ and the corresponding CI. Consider the penalized log-likelihood function of $\beta_{c}$ as follows: $pl=-{\left({\hat{\beta }}_{c}-\beta_{c}\right)}^{2}/\left(2\hat{\mathrm{Var}}\left({\hat{\beta }}_{c}\right)\right)+\lambda \log\left|\beta_{c}\right|$, where λ>0 is a penalty parameter and is taken to be $Z_{1-\alpha /2}^{2}/4$ as suggested by Wang et al. [41] because the CI obtained by the PF method is always bounded with $\lambda =Z_{1-\alpha /2}^{2}/4$. By maximizing the function pl, we have the penalized denominator ${\tilde{\beta }}_{c}={\hat{\beta }}_{c}/2+\mathrm{sign}\left({\hat{\beta }}_{c}\right)\sqrt{{\hat{\beta }}_{c}^{2}/4+\lambda \hat{\mathrm{Var}}\left({\hat{\beta }}_{c}\right)}$, where sign(·) is the signum function. Further, we can get $\hat{\mathrm{Var}}\left({\tilde{\beta }}_{c}\right)=\xi^{2}\hat{\mathrm{Var}}\left({\hat{\beta }}_{c}\right)+O\left(n^{-3}\right)$, where $\xi ={\tilde{\beta }}_{c}/\left(2{\tilde{\beta }}_{c}-{\hat{\beta }}_{c}\right)$. If we replace ${\hat{\beta }}_{c}$ by ${\tilde{\beta }}_{c}$ to obtain the point estimate $\tilde{\gamma }={\hat{\beta }}_{c}^{\left(1\right)}/{\tilde{\beta }}_{c}$, then $\tilde{\gamma }$ is a biased estimate of γ. To reduce this bias, we need to correct the numerator ${\hat{\beta }}_{c}^{\left(1\right)}$ by ${\tilde{\beta }}_{c}^{\left(1\right)}={\hat{\beta }}_{c}^{\left(1\right)}+\tilde{\gamma }\left({\tilde{\beta }}_{c}-{\hat{\beta }}_{c}\right)$. Correspondingly, we can get $\hat{\mathrm{Var}}\left({\tilde{\beta }}_{c}^{\left(1\right)}\right)=\xi^{-2}\hat{\mathrm{Var}}\left({\hat{\beta }}_{c}^{\left(1\right)}\right)-4\left(\xi^{-1}-1\right)\tilde{\gamma }\hat{\mathrm{Cov}}\left({\hat{\beta }}_{c}^{\left(1\right)},{\hat{\beta }}_{c}\right)+4{\left(1-\xi \right)}^{2}{\tilde{\gamma }}^{2}\hat{\mathrm{Var}}\left({\hat{\beta }}_{c}\right)$ and $\hat{\mathrm{Cov}}\left({\tilde{\beta }}_{c}^{\left(1\right)},{\tilde{\beta }}_{c}\right)=\hat{\mathrm{Cov}}\left({\hat{\beta }}_{c}^{\left(1\right)},{\hat{\beta }}_{c}\right)-2\xi \left(1-\xi \right)\tilde{\gamma }\hat{\mathrm{Var}}\left({\hat{\beta }}_{c}\right)$. After obtaining the corrected denominator ${\tilde{\beta }}_{c}$ and the corrected numerator ${\tilde{\beta }}_{c}^{\left(1\right)}$, ${\hat{\gamma }}^{*}={\tilde{\beta }}_{c}^{\left(1\right)}/{\tilde{\beta }}_{c}$ truncated by [0, 2] is the penalized point estimate of γ, which is denoted by ${\hat{\gamma }}_{GPF}$. The construction process of the corresponding CI of ${\hat{\gamma }}_{GPF}$ is the same as the Fieller’s method, except for respectively replacing ${\hat{\beta }}_{c}$, ${\hat{\beta }}_{c}^{\left(1\right)}$, $\hat{\mathrm{Var}}\left({\hat{\beta }}_{c}\right)$, $\hat{\mathrm{Var}}\left({\hat{\beta }}_{c}^{\left(1\right)}\right)$ and $\hat{\mathrm{Cov}}\left({\hat{\beta }}_{c}^{\left(1\right)},{\hat{\beta }}_{c}\right)$ by ${\tilde{\beta }}_{c}$, ${\tilde{\beta }}_{c}^{\left(1\right)}$, $\hat{\mathrm{Var}}\left({\tilde{\beta }}_{c}\right)$, $\hat{\mathrm{Var}}\left({\tilde{\beta }}_{c}^{\left(1\right)}\right)$ and $\hat{\mathrm{Cov}}\left({\tilde{\beta }}_{c}^{\left(1\right)},\;{\tilde{\beta }}_{c}\right)$ in Equation (6). However, it should be noted that although the CI of γ based on the PF method is always bounded when $\lambda =Z_{1-\alpha /2}^{2}/4$, it may be out of [0, 2] and we need to truncate it by [0, 2].

### 2.4. Point Estimate and Credible Interval of γ by Bayesian Method

Note that the point estimates (${\hat{\gamma }}_{GF}$ and ${\hat{\gamma }}_{GPF}$), and the corresponding CIs mentioned above, are cut off in the interval [0, 2] and cannot directly incorporate the information on γ∈[0, 2]. Therefore, in this subsection, we introduce the Bayesian method to give the point estimate and the credible interval of γ by considering the prior information of γ∈[0, 2]. Specifically, we have the posterior distribution of the unknown parameter $\theta_{.}$ as follows

$$f\left(\theta_{.}|Y,X^{\left(1\right)},\;X^{\left(2\right)},Z\right)=\frac{f\left(\theta_{.}\right)L_{.}\left(\theta_{.}\right)}{\int f\left(\theta_{.}\right)L_{.}\left(\theta_{.}\right)d\theta_{.}},$$

where $f\left(\theta_{.}\right)$ is the joint prior distribution of $\theta_{.}$; when the traits are quantitative, $\theta_{.}=\theta_{1}$ and $L_{.}\left(\theta_{.}\right)=L_{1}\left(\theta_{1}\right)$; when the traits are qualitative, $\theta_{.}=\theta_{2}$ and $L_{.}\left(\theta_{.}\right)=L_{2}\left(\theta_{2}\right)$. However, in general, we cannot get the analytical solutions of $f\left(\theta_{.}|Y,X^{\left(1\right)},\;X^{\left(2\right)},Z\right)$. Therefore, it is not feasible to directly sample from the posterior distribution. Fortunately, there are several algorithms for sampling from an approximate distribution of the posterior distribution, such as the Hamiltonian Monte Carlo (HMC) algorithm which can be implemented by the “rstan” package in R [43]. On the other hand, according to Annis et al. [43], the correlation between the parameters has little influence on the HMC algorithm. To simplify the operations, and improve the sampling efficiency, we assume that the unknown parameters in $\theta_{.}$ are independent of each other, and use the HMC algorithm to sample from the approximate posterior distribution of $\theta_{.}$. In other words, we choose the prior distribution for each unknown parameter separately.

The prior distributions of the parameters in $\theta_{.}$ are selected as follows. To reduce the influence of the selection of the prior distributions on the results, for nuisance parameters $\beta_{0}$, $\beta_{c}$ and b (there is an additional nuisance parameter σ when the trait is quantitative), we choose the weak prior distributions [44]. Specifically, we assume that the prior distributions of $\beta_{0}$ and $\beta_{c}$ are both $\;N\left(0,10^{2}\right)$, and that of b is $MVN\left(0,\;\mathrm{diag}\left(10^{2},\;10^{2},\;\ldots ,\;10^{2}\right)\right)$. For quantitative traits, we also specify the prior distribution of σ to be an exponential distribution, i.e., σ~exp(1). As for the parameter γ of interest, which is used to measure the mean degree of the skewness of the XCI over all the SNPs in the gene and satisfies the constraint condition of γ∈[0, 2], we consider two possible prior distributions. The first one is the truncated normal distribution, with both parameters being 1 and the values ranging from 0 to 2, and the probability density function of the prior distribution is

$$f\left(\gamma \right)=\{\begin{array}{cc} \frac{\phi \left(\gamma -1\right)}{\frac{1}{\sqrt{2\pi }}\int_{0}^{2}\exp\left[-\frac{1}{2}{\left(x-1\right)}^{2}\right]dx}, & 0\leq \gamma \leq 2\\ 0, & \mathrm{otherwise} \end{array},$$

where ϕ(·) is the probability density function of the standard normal distribution. In this way, γ not only satisfies the constraint condition of γ∈[0, 2], but also the probability of γ being close to 1 is the highest, which is consistent with the literature [2], i.e., most of the SNPs on the X chromosome undergo the XCI-R. Meanwhile, the selected truncated normal distribution of γ also avoids that the probability of γ taking the extreme value (0 or 2) is too low, which may be more suitable for practical applications. The second prior distribution of γ is a uniform distribution, i.e., γ~U(0, 2).

After specifying the prior distributions of all the unknown parameters, we can get enough samples of γ through the HMC algorithm, and then calculate the mode of the samples as the point estimate of γ, and the highest posterior density interval (HPDI) as the credible interval of γ. Here, we denote the Bayesian methods with the truncated normal prior and the uniform prior as GBN and GBU, and the point estimates obtained by these two methods are denoted as ${\hat{\gamma }}_{GBN}$ and ${\hat{\gamma }}_{GBU}$, respectively.

## 3. Results

### 3.1. Simulation Settings

We conducted extensive simulation studies to evaluate the performances of the proposed point estimation and interval estimation methods. The number of female subjects (i.e., the sample size n) is set to be 500 and 2000. Consider a gene associated with the trait under study and the number of the SNPs in the gene (i.e., J) is fixed at 100, i.e., we assume that all the 100 SNPs are associated with the trait. Meanwhile, we define η as the proportion of rare variants among the 100 SNPs. To explore the effect of η on the proposed methods, we set η=0, 0.4 and 1, which correspond to the cases of all the 100 SNPs only including common variants, the 100 SNPs simultaneously containing common and rare variants, and all the 100 SNPs only consisting of rare variants, respectively. Among them, the MAFs for common variants are sampled from U(0.01, 0.5), while the MAFs for rare variants are randomly simulated from U(0.005, 0.01) [45,46,47]. We generate the genotypes of n female subjects by referring to the ideas of Wang et al. [45], Basu et al. [46], and Turkmen et al. [47]. We first generate a latent vector $V={\left(V_{1},V_{2},\ldots ,V_{100}\right)}^{T}$ from the multivariate normal distribution with the mean vector being 0 and the elements of the variance-covariance matrix satisfying $\mathrm{Var}(V_{j})=1$ and $\mathrm{Corr}\left(V_{j},V_{k}\right)=\rho^{\left|j-k\right|}$ (j, k=1, 2, …, 100) [45,47], where the linkage disequilibrium among the SNPs is taken into consideration. For simplicity, we set ρ=0.5 in our simulation studies. Once V is generated, it is then transformed to 0 (major allele) or 1 (minor allele) determined by the corresponding MAFs. This process is repeated twice, and two simulated vectors of length 100 are put together to form the genotypes at the 100 SNPs for a female subject. After simulating the genotypes of n female subjects, we have an n×100 genotypic value matrix$\;G={\left(G_{1},\;G_{2},\;\ldots ,\;G_{n}\right)}^{T}$ with the elements being 0, 1 or 2, and then we replace the elements of G equal to 1 with γ to simulate the information on the XCI-S. Note that to simplify the simulation and better evaluate the performances of our proposed methods (e.g., the calculation of the mean squared errors (MSEs) of the point estimates requires a single true value of γ for each replicate; the details are given later), we set the degrees of the XCI skewing $\gamma_{j}$’s at all the 100 SNPs to be the same in the simulation study (i.e., $\gamma_{j}=\gamma ,\;j=1,\;2,\;\ldots ,\;100$).

We only consider a covariate Q, which is generated from the standard normal distribution. For the quantitative trait, we simulate the trait value $y_{i}$ of the ith female according to the following model

$$y_{i}=\beta_{0}+\beta_{1}g_{i1}+\beta_{2}g_{i2}+\ldots +\beta_{100}g_{i100}+\delta Q_{i}+\varepsilon_{i},$$

where $\varepsilon_{i}$ is the random error, which is generated from the standard normal distribution; $\beta_{0}$ is the intercept and δ is the regression coefficient of the covariate Q, and both the parameters are set to be 0.5 [36]; $\left|\beta_{j}\right|=e\left|\log_{10}{\mathrm{MAF}}_{j}\right|/2$ is the regression coefficient of the genotypic value $g_{ij}$ at the jth SNP [33,34,36], where e is the tuning parameter and is used to avoid the effect of a SNP being too large or too small [36]. To highlight the effects of rare variants on the trait, we set e=1.5 when the jth SNP has a rare variant, otherwise e=1.1. Further, notice that the directions of the effects of different SNPs on the trait may be different. Therefore, we consider the proportion of the SNPs with positive effects among the 100 SNPs (denoted by τ) and set τ to be 0.6 and 1, indicating that the effect directions of some SNPs are positive and some are negative, and all the SNP effects are positive, respectively. We do not simulate the case of τ=0 (i.e., all the SNP effects are negative) because the results with τ=0 are similar to those with τ=1. As for the qualitative trait, the model for generating the affection status $y_{i}$ of the ith female is as follows

$$\mathrm{Logit}\left(Pr\left(y_{i}=1|G_{i},Q_{i}\right)\right)=\beta_{0}+\beta_{1}g_{i1}+\beta_{2}g_{i2}+\ldots +\beta_{100}g_{i100}+\delta Q_{i}.$$

All of the parameters are the same as when simulating the quantitative trait, except that we need to set the case-control ratio to be 1:1.

After simulating the genotypes and the trait values, we use model (4) to obtain the estimates of $\beta_{c}^{\left(1\right)}$ and $\beta_{c}^{\left(2\right)}$, where $X_{i}^{\left(1\right)}=\sum_{j=1}^{100}\omega_{j}g_{ij}^{\left(1\right)}$, $X_{i}^{\left(2\right)}=\sum_{j=1}^{100}\omega_{j}g_{ij}^{\left(2\right)}$, $\omega_{j}=Beta\left({\hat{\mathrm{MAF}}}_{j},\;0.5,\;0.5\right)$, and ${\hat{\mathrm{MAF}}}_{j}$ is the estimate of the MAF at the jth SNP. Then, we get the point estimate ${\hat{\gamma }}_{GF}$, the penalized point estimate ${\hat{\gamma }}_{GPF}$, and the CIs of γ derived by the Fieller’s and the PF methods. As for the Bayesian methods, the HMC algorithm is implemented through the “sampling” function in the R package “rstan”. We set 8 chains for the parallel sampling in the simulation. For each chain, we extract 10,000 samples, and the first 5000 are used for warm-up. So, we finally get 40,000 samples. To ensure the convergence, the target acceptance rate is set to be 0.99.

The above simulation steps are all implemented in the R software (version 4.1.1, http://r-project.org, accessed on 5 January 2022). For each simulation setting, the number of the replicates is fixed to be 500, and for each replicate, the true value of γ is sampled from the uniform distribution U(0, 2). To evaluate the accuracy and the robustness of ${\hat{\gamma }}_{GBN}$, ${\hat{\gamma }}_{GBU}$, ${\hat{\gamma }}_{GPF}$ and ${\hat{\gamma }}_{GF}$, we calculate the MSEs of these point estimates. Here, $\mathrm{MSE}=\overset{500}{\underset{s=1}{\sum }}{\left({\hat{\gamma }}_{s}-\gamma_{s}\right)}^{2}/500$, where $\gamma_{s}$ represents the true value of γ and ${\hat{\gamma }}_{s}$ is the point estimate in the sth replicate (s=1, 2, …, 500). Note that ${\hat{\gamma }}_{GBN}$ and ${\hat{\gamma }}_{GBU}$ are always between 0 and 2, so we only compute the proportions of ${\hat{\gamma }}_{GPF}$ and ${\hat{\gamma }}_{GF}$ taking the extreme values (0 or 2), respectively. Meanwhile, scatter plots are used to show the relationship between the four point estimates and the true values of γ. To compare the performances of the GBN, GBU, PF and Fieller’s methods in the interval estimation, we calculate the CP as well as the mean, the median, the standard deviation and the interquartile range of the widths of the 95% HPDIs or CIs (denoted by $W_{mean}$, $W_{median}$, $W_{sd}$ and $W_{iqr}$), respectively. For the PF and Fieller’s methods, we also compute the proportions of the empty sets (EP), the noninformative intervals (NP), and the discontinuous intervals (DP) to further compare the effectiveness of these two methods, where the noninformative interval means the CI being [0, 2]. However, it should be noted that the GBN and GBU methods avoid the cases of empty sets, noninformative intervals, and discontinuous intervals occurring. In addition, we draw the scatter plots between the interval widths of the four proposed methods and the true values of γ.

### 3.2. Simulation Results

The proportions of the extreme values (0 or 2) for ${\hat{\gamma }}_{GPF}$ and ${\hat{\gamma }}_{GF}$ are shown in Table 1. It can be seen from the table that the proportions of the point estimates equal to 0 are the same for both ${\hat{\gamma }}_{GPF}$ and ${\hat{\gamma }}_{GF}$, while the proportion of the point estimates equal to 2 for ${\hat{\gamma }}_{GPF}$ is reduced. This is because before the truncation, both ${\hat{\gamma }}^{*}={\tilde{\beta }}_{c}^{\left(1\right)}/{\tilde{\beta }}_{c}$ and $\hat{\gamma }={\hat{\beta }}_{c}^{\left(1\right)}/{\hat{\beta }}_{c}$ always have the same sign, and ${\hat{\gamma }}^{*}$ is bounded. Specifically, when ${\hat{\gamma }}^{*}$ and $\hat{\gamma }$ are negative, ${\hat{\gamma }}_{GPF}$ and ${\hat{\gamma }}_{GF}$ are both 0. On the other hand, when ${\hat{\gamma }}^{*}$ and $\hat{\gamma }$ are positive, compared with $\hat{\gamma }$, the proportion of ${\hat{\gamma }}^{*}$ being greater than 2 decreases. Further, from Table 1, with the increase of the sample size or the trait changing from qualitative to quantitative, the proportions of the extreme values for ${\hat{\gamma }}_{GPF}$ and ${\hat{\gamma }}_{GF}$ both become less. Next, let us take a look at the effects of the proportion of the rare variants (η) and the proportion of the SNPs with the positive effects (τ) among all the SNPs on the proportions of the extreme values for ${\hat{\gamma }}_{GPF}$ and ${\hat{\gamma }}_{GF}$. Under the situation that the trait is quantitative and τ=0.6 (i.e., the effect directions of some SNPs are positive and some are negative), the proportions of the extreme values (0 and 2) for ${\hat{\gamma }}_{GPF}$ and ${\hat{\gamma }}_{GF}$ with η=0 (all the SNPs only include common variants) are less than those with η=1 (all the SNPs only consist of rare variants), irrespective of the sample size (n). As for the qualitative trait, when n=2000 and τ=0.6, the proportion of the extreme values equal to 0 for ${\hat{\gamma }}_{GPF}$ and the proportions of the extreme values (0 and 2) for ${\hat{\gamma }}_{GF}$ with η=0 are smaller than those with η=1, while the proportion of the extreme values equal to 2 for ${\hat{\gamma }}_{GPF}$ with η=0 (12.8%) is larger than that with η=1 (10.4%). When the trait is qualitative, n=500 and τ=0.6, the results are similar to those with n=2000, except that the proportion of the extreme values equal to 2 for ${\hat{\gamma }}_{GF}$ with η=0 (20.0%) and that with η=1 (19.2%) are very close to each other. In addition, the proportions of the extreme values (0 or 2) for ${\hat{\gamma }}_{GPF}$ and ${\hat{\gamma }}_{GF}$. have no obvious trends for other cases of different values of η and τ.

The MSEs of the four point estimates (${\hat{\gamma }}_{GBN}$, ${\hat{\gamma }}_{GBU}$, ${\hat{\gamma }}_{GPF}$ and ${\hat{\gamma }}_{GF}$) are listed in Table 2. From Table 2, we can see that the MSEs of ${\hat{\gamma }}_{GBN}$ and ${\hat{\gamma }}_{GBU}$ are smaller than those of ${\hat{\gamma }}_{GPF}$ and ${\hat{\gamma }}_{GF}$, and the MSE of ${\hat{\gamma }}_{GBN}$ is the smallest. When the sample size increases or the trait turns from qualitative to quantitative, the MSEs of these four point estimates decrease significantly. In general, the MSEs of the four point estimates gradually become larger when η changes from 0, 0.4 to 1 (i.e., higher proportion of rare variants) and other parameters are kept unchanged, except for the case when the trait is quantitative, n=500 and τ=1. On the other hand, the MSEs of the four point estimates with τ=0.6 (i.e., the effect directions of some SNPs are positive and some are negative) are smaller than those with τ=1 (i.e., all the SNP effects are positive), when other parameters are fixed.

**Figure 1:**
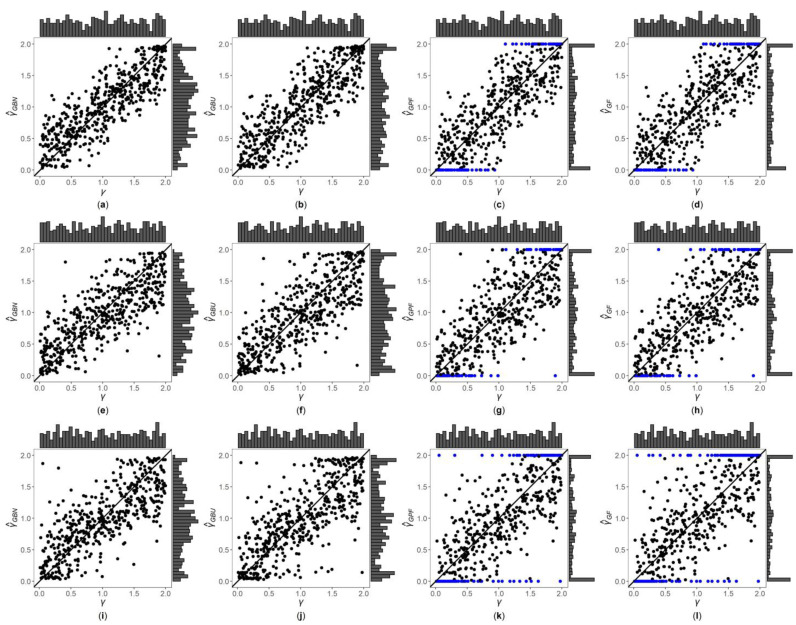
Scatter plots of point estimates of γ against true values of γ for quantitative trait with n=500 and τ=0.6. The blue points represent the extreme values (0 or 2). (**a**) ${\hat{\gamma }}_{GBN}$ with η=0; (**b**) ${\hat{\gamma }}_{GBU}$ with η=0; (**c**) ${\hat{\gamma }}_{GPF}$ with η=0; (**d**) ${\hat{\gamma }}_{GF}$ with η=0; (**e**) ${\hat{\gamma }}_{GBN}$ with η=0.4; (**f**) ${\hat{\gamma }}_{GBU}$ with η=0.4; (**g**) ${\hat{\gamma }}_{GPF}$ with η=0.4; (**h**) ${\hat{\gamma }}_{GF}$ with η=0.4; (**i**) ${\hat{\gamma }}_{GBN}$ with η=1; (**j**) ${\hat{\gamma }}_{GBU}$ with η=1; (**k**) ${\hat{\gamma }}_{GPF}$ with η=1; (**l**) ${\hat{\gamma }}_{GF}$ with η=1.

**Figure 2:**
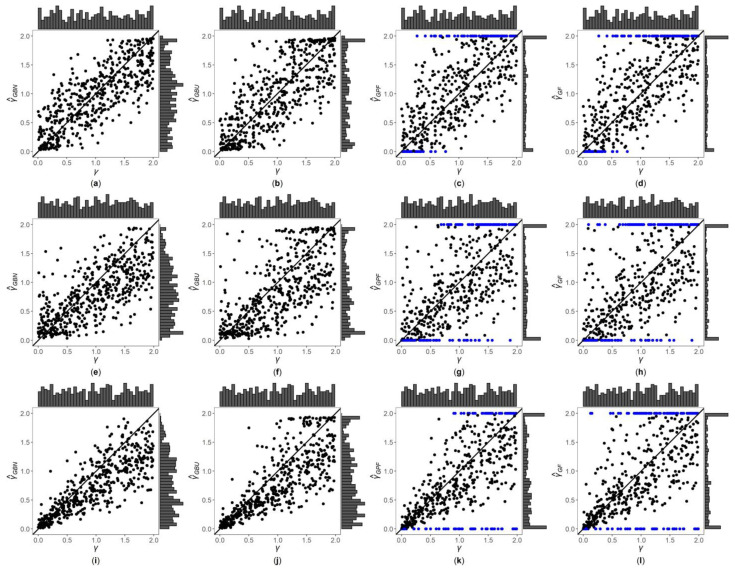
Scatter plots of point estimates of γ against true values of γ for quantitative trait with n=500 and τ=1. The blue points represent the extreme values (0 or 2). (**a**) ${\hat{\gamma }}_{GBN}$ with η=0; (**b**) ${\hat{\gamma }}_{GBU}$ with η=0; (**c**) ${\hat{\gamma }}_{GPF}$ with η=0; (**d**) ${\hat{\gamma }}_{GF}$ with η=0; (**e**) ${\hat{\gamma }}_{GBN}$ with η=0.4; (**f**) ${\hat{\gamma }}_{GBU}$ with η=0.4; (**g**) ${\hat{\gamma }}_{GPF}$ with η=0.4; (**h**) ${\hat{\gamma }}_{GF}$ with η=0.4; (**i**) ${\hat{\gamma }}_{GBN}$ with η=1; (**j**) ${\hat{\gamma }}_{GBU}$ with η=1; (**k**) ${\hat{\gamma }}_{GPF}$ with η=1; (**l**) ${\hat{\gamma }}_{GF}$ with η=1.

Figure 1, Figure 2 and Figures S1–S6 are the scatter plots of the four point estimates against the true values of γ under different simulation settings. These figures can more intuitively compare the performances of the four point estimates. For example, Figure 1 and Figure 2 are the scatter plots of the four point estimates against the true values of γ for the quantitative trait with n=500, and τ=0.6 and 1, respectively. In each figure, subplots (a)–(d) (four subplots in the first row) are respectively the scatter plots of ${\hat{\gamma }}_{GBN}$, ${\hat{\gamma }}_{GBU}$, ${\hat{\gamma }}_{GPF}$ and ${\hat{\gamma }}_{GF}$ with η=0; subplots (e)–(h) (four subplots in the second row) and subplots (i)–(l) (four subplots in the third row) are the corresponding scatter plots with η=0.4 and 1, respectively. By comparing the four subplots in the same row of each figure, we find that the two point estimates (${\hat{\gamma }}_{GBN}$ and ${\hat{\gamma }}_{GBU}$) obtained by the Bayesian methods are closer to the true values of γ, and both perform better than ${\hat{\gamma }}_{GPF}$ and ${\hat{\gamma }}_{GF}$. On the other hand, note that the distribution of the true value of γ is U(0, 2), and it can be seen from the figures that the distributions of ${\hat{\gamma }}_{GBN}$ and ${\hat{\gamma }}_{GBU}$ are more uniform, while the distributions of ${\hat{\gamma }}_{GPF}$ and ${\hat{\gamma }}_{GF}$ are skewed towards the extreme values (0 and 2). Meanwhile, by respectively comparing subplots (a), (e) and (i) for ${\hat{\gamma }}_{GBN}$ with subplots (b), (f) and (j) for ${\hat{\gamma }}_{GBU}$, there is a little greater dispersion for ${\hat{\gamma }}_{GBU}$ than ${\hat{\gamma }}_{GBN}$. In addition, from subplots (c), (g) and (k) for ${\hat{\gamma }}_{GPF}$ and subplots (d), (h) and (l) for ${\hat{\gamma }}_{GF}$, we observe that there exist many extreme point estimates for ${\hat{\gamma }}_{GPF}$ and ${\hat{\gamma }}_{GF}$ (represented by the blue points). Moreover, the scatter plots for ${\hat{\gamma }}_{GPF}$ and ${\hat{\gamma }}_{GF}$ provide the additional information that most of the extreme point estimates generally occur when the true values of γ are less than 0.5 or greater than 1.5. Further, by comparing the subplots in different rows of each figure when τ=0.6 (Figure 1, Figures S1, S3 and S5), i.e., η changing from 0, 0.4 to 1, the dispersions of the four point estimates generally increase, indicating that, in general, the MSEs of the four point estimates become larger, which are consistent with the results in Table 2. The numbers of the blue points in subplots (c) and (d) with η=0 are much less than those in subplots (k) and (l) with η=1, respectively. However, for those figures with τ=1 (Figure 2, Figure S2, S4 and S6), there is no obvious trend for the number of the blue points. Compared to Figure 1 (τ=0.6), the agreements between the four point estimates and the true values of γ in Figure 2 (τ=1) are worse, which can also be seen in other figures (Figures S1, S3 and S5 vs. Figures S2, S4 and S6, respectively). Observing Figure 2, we find that the four point estimation methods may underestimate γ when τ=1. Finally, these four point estimation methods perform better for the quantitative trait than for the qualitative trait (Figure 1, Figure 2, Figures S1 and S2 vs. Figures S3–S6, respectively), and when the sample size increases (Figures S1, S2, S5 and S6 vs. Figure 1, Figure 2, Figures S3 and S4, respectively).

Table 3 displays the EPs, NPs and DPs of the PF and Fieller’s methods. From Table 3, we observe that the EPs of the PF method are generally smaller than, or equal to, those of the Fieller’s method, except for the quantitative trait with n=500, η=0.4 or 1, and τ=1, and the qualitative trait with n=500 or 2000, η=0.4 or 1, and τ=1. However, the NPs of the PF method are always smaller than, or equal to, those of the Fieller’s method. Note that when we use the PF and Fieller’s methods to calculate the CIs of γ, we need to truncate the CIs by the interval [0, 2]. As such, compared to the Fieller’s method, the PF method can get shorter CIs, which means that the PF method reduces the possibility of the truncated CIs being the noninformative intervals. On the other hand, if the CIs before the truncation are disjoint from the interval [0, 2], the PF method will increase the possibility that the truncated CIs are empty sets, which is the reason why the PF method may have bigger EPs than the Fieller’s method in some scenarios. In addition, all the DPs of the PF method are equal to 0. This is because we consider the penalty parameter $\lambda =Z_{1-\alpha /2}^{2}/4$, and the CIs derived by the PF method are always continuous. With increase of the sample size, the NPs of the PF and Fieller’s methods and the DPs of the Fieller’s method become smaller. Moreover, under the same simulation settings, the NPs of both methods, and the DPs of the Fieller’s method, for the quantitative trait are less than those for the qualitative trait. Under the situation that τ=0.6, when η changes from 0, 0.4 to 1 and other parameters are kept unchanged, the EPs of both methods have no obvious trends, while the NPs of both methods and the DPs of the Fieller’s method generally become larger. As for τ=1, when η changing from 0, 0.4 to 1 and other parameters being fixed, the EPs of the PF method appear larger except for the quantitative trait and n=2000, while the DPs of the Fieller’s method are relatively stable, and the NPs of the PF and Fieller’s methods show a trend of first increasing and then decreasing on most occasions. On the other hand, when other parameters are fixed, the EPs and NPs of the PF and Fieller’s methods with τ=0.6 are smaller than those with τ=1 in most cases, and the DPs of the Fieller’s method with τ=0.6 are larger than or equal to those with τ=1.

The CPs, $W_{mean}$ and $W_{median}$ of the GBN, GBU, PF and Fieller’s methods are displayed in Table 4, and the corresponding $W_{sd}$ and $W_{iqr}$ are given in Table 5. Table 4 demonstrates that, for the quantitative trait, the CPs of the GBN, GBU and Fieller’s methods are controlled around 95%. However, when n=500, η=1 and τ=1, the CP of the PF method is underestimated (87.8%). As the sample size increases to 2000 and other parameters remain unchanged, the CP of the PF method is 96.6%. For the qualitative trait, when n=500, the CPs of the GBN, GBU and PF methods are underestimated in most situations. With the increase of the sample size to 2000, the CPs of these three methods generally increase to be around 95%, but the CPs when η=1 and τ=1 are still underestimated. Thus, for this simulation setting, we conduct an additional simulation study with larger sample sizes (3000 and 4000), and the corresponding results are presented in Table S1. It is shown in Table S1 that the CPs of these three methods are closer to 95% when the sample size continues to increase. This is explainable by the fact that qualitative traits generally require larger samples to achieve the same CPs than quantitative traits. In addition, we can see from Table 4 that the Fieller’s method has higher CPs under various simulation settings for the qualitative trait. However, according to Table 3, when the trait is qualitative, the NPs of the Fieller’s method are relatively high, which means that many CIs obtained by the Fieller’s method are the noninformative intervals (i.e., [0, 2]). This may explain why the CPs of the Fieller’s method are on the high side. Further, from Table 4 and Table 5, the $W_{mean}$, $W_{median}$, $W_{sd}$ and $W_{iqr}$ of the GBN and GBU methods are smaller than those of the PF and Fieller’s methods in most situations. The GBN method has the smallest $W_{mean}$, $W_{median}$ and $W_{iqr}$ in most cases, and it also has the smallest $W_{sd}$ under all the simulated settings. As can be seen from Table 4, when the trait is qualitative and n=500, the $W_{median}$’s of the Fieller’s method are all 2, which indicates that in this case, more than half of the CIs based on the Fieller’s method are the noninformative intervals. This is consistent with the results of the NPs in Table 3. When the sample size increases, or the trait turns from qualitative into quantitative, the $W_{mean}$’s and $W_{median}$’s of the four interval estimation methods greatly decrease. However, for the $W_{sd}$ and $W_{iqr}$, there are different trends in some situations. For example, when the trait is qualitative, the $W_{sd}$’s and $W_{iqr}$’s of the four methods become larger in most cases as the sample size increases. Note that the widths of the intervals obtained by the four methods are closer to 2 and the corresponding variation will be smaller when n=500. With the sample size increasing, the widths of the intervals gradually decrease and the corresponding variation appears larger, which may cause the bigger $W_{sd}$ and $W_{iqr}$.

In the case of τ=0.6, the four methods have larger $W_{mean}$’s and $W_{median}$’s in most cases when η changes from 0, 0.4 to 1, while for the scenario of τ=1, the four methods show a trend of first increasing and then decreasing on most occasions, except that the $W_{mean}$’s and $W_{median}$’s of the Fieller’s method are gradually larger for the qualitative trait. When the trait is quantitative and τ=0.6, the $W_{sd}$’s and $W_{iqr}$’s of the four methods become larger with η increasing from 0, 0.4 to 1, irrespective of the sample size. When the trait is qualitative, n=500 and τ=0.6, as η is bigger, the $W_{sd}$’s and $W_{iqr}$’s of the four methods generally are smaller, while when n=2000, the $W_{sd}$’s of the four methods and the $W_{iqr}$’s of the GBN and GBU methods are relatively stable, and the $W_{iqr}$’s of the PF and Fieller’s methods generally become larger. For the quantitative trait with n=500 and τ=1, with the increase of η, the $W_{sd}$’s and $W_{iqr}$’s of the GBN, GBU and Fieller’s methods appear smaller and those of the PF method are larger in most situations, while in the case of n=2000, the four methods usually have larger $W_{sd}$’s and $W_{iqr}$’s. When the trait is qualitative and τ=1, with η increasing, the $W_{sd}$’s and $W_{iqr}$’s of the GBN and GBU methods present a tendency of first decreasing and then increasing on most occasions, while those of the PF method are larger in most cases, and those of the Fieller’s method become smaller, irrespective of the sample size. On the other hand, when other parameters are fixed, the $W_{mean}$’s and $W_{median}$’s of the four methods with τ=0.6 are smaller than those with τ=1, except for the $W_{mean}$’s of the GBN, GBU and PF methods and the $W_{median}$’s of the GBN and GBU methods for the quantitative trait with n=500 and η=1, and those for the qualitative trait with n=500 or 2000, and η=1. Under the scenarios where η is kept unchanged, the $W_{sd}$’s and $W_{iqr}$’s of the GBN, GBU and Fieller’s methods with τ=0.6 are generally larger than those with τ=1 for the quantitative trait with n=500, and the qualitative trait with n=500 or 2000, while there are different trends for the quantitative trait with n=2000. In addition, the $W_{sd}$’s and $W_{iqr}$’s of the PF method with τ=0.6 generally are smaller than those with τ=1, when other parameters are fixed.

**Figure 3:**
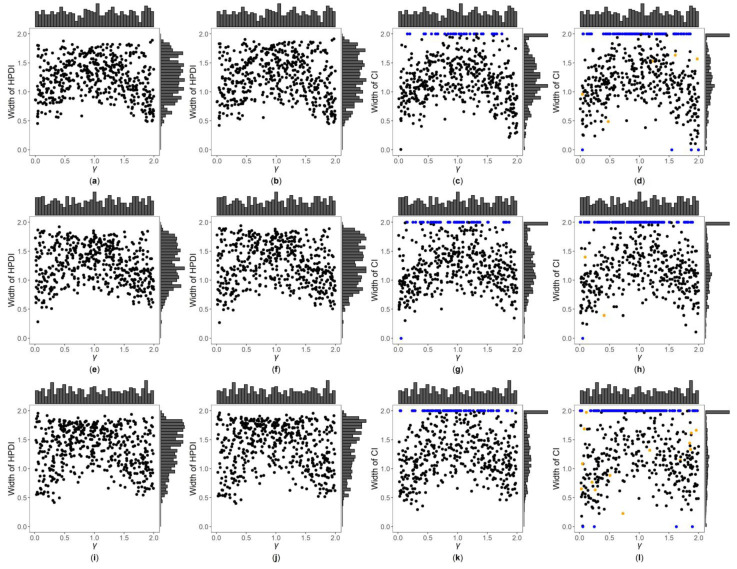
Widths of highest posterior density intervals (HPDIs) or confidence intervals (CIs) of GBN, GBU, PF and Fieller’s methods against true values of γ for quantitative trait with n=500 and τ=0.6. The blue points represent the widths of the empty sets or the noninformative intervals, and the orange points represent the widths of the discontinuous intervals. (**a**) GBN with η=0; (**b**) GBU with η=0; (**c**) PF with η=0; (**d**) Fieller with η=0; (**e**) GBN with η=0.4; (**f**) GBU with η=0.4; (**g**) PF with η=0.4; (**h**) Fieller with η=0.4; (**i**) GBN with η=1; (**j**) GBU with η=1; (**k**) PF with η=1; (**l**) Fieller with η=1.

**Figure 4:**
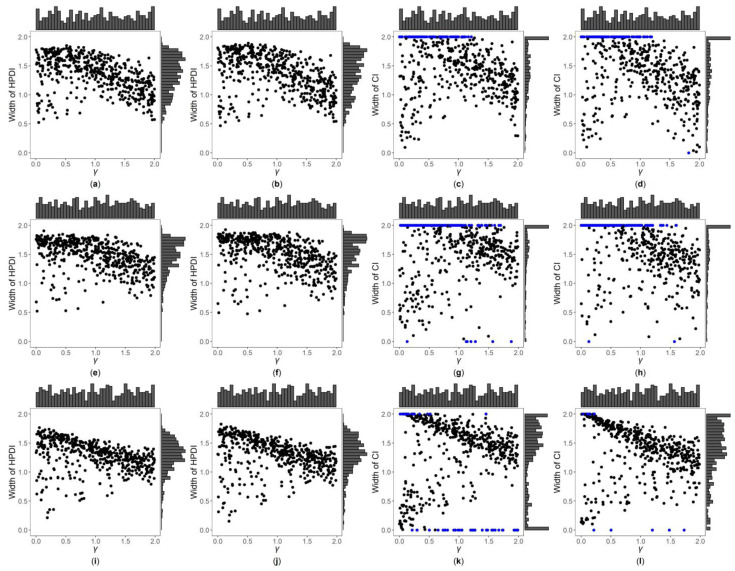
Widths of HPDIs or CIs of GBN, GBU, PF and Fieller’s methods against true values of γ for quantitative trait with n=500 and τ=1. The blue points represent the widths of the empty sets or the noninformative intervals. (**a**) GBN with η=0; (**b**) GBU with η=0; (**c**) PF with η=0; (**d**) Fieller with η=0; (**e**) GBN with η=0.4; (**f**) GBU with η=0.4; (**g**) PF with η=0.4; (**h**) Fieller with η=0.4; (**i**) GBN with η=1; (**j**) GBU with η=1; (**k**) PF with η=1; (**l**) Fieller with η=1.

Figure 3, Figure 4 and Figures S7–S12 are the scatter plots of the widths of the 95% HPDIs or CIs obtained by the four interval estimation methods (GBN, GBU, PF and Fieller) against the true values of γ under different simulation settings. We can clearly observe the distributions of the widths of the HPDIs or CIs through these figures. For example, Figure 3 and Figure 4 are the scatter plots of the widths of the HPDIs or CIs against the true values of γ for the quantitative trait with n=500, and τ=0.6 and 1, respectively. In each figure, subplots (a)–(d) (four subplots in the first row) are respectively the scatter plots for the GBN, GBU, PF and Fieller’s methods with η=0; subplots (e)–(h) (four subplots in the second row) and subplots (i)-(l) (four subplots in the third row) are the corresponding scatter plots with η=0.4 and 1, respectively. It can be seen from the four subplots in the same row of each figure that the distributions of the widths of the HPDIs for the GBN and GBU methods are similar, and both have smaller dispersions than those of the CIs for the PF and Fieller’s methods. Furthermore, these figures display that the distributions of the interval widths for the PF and Fieller’s methods are greatly more skewed towards 2 than the GBN and GBU methods. We respectively compare subplots (a), (e) and (i) for the GBN method with subplots (b), (f) and (j) for the GBU method and find that the dispersions of the widths of the HPDIs for the GBN method are slightly smaller than the GBU method. Additionally, subplots (c), (g) and (k) for the PF method, and subplots (d), (h) and (l) for the Fieller’s method, show that the PF and Fieller’s methods may yield empty sets or noninformative intervals (displayed by the blue points), and the Fieller’s method may also get discontinuous intervals (shown by the orange points). By comparing the subplots in different rows of each figure (Figure 3 and Figure S7) when the trait is quantitative and τ=0.6, the dispersions of the widths of the HPDIs or CIs become slightly larger as η changing from 0, 0.4 to 1, and it can also be seen from Figure 3 that the distributions of the interval widths are a little more skewed towards 2. On the other hand, when the trait is qualitative with τ=0.6 (Figures S9 and S11), there are no obvious trends in the dispersions of the interval widths, except that their distributions are more skewed towards 2. However, under the situation that τ=1 (Figure 4, Figures S8, S10 and S12), the points in these figures become less discrete in most cases when η increases, and the overall widths of the four interval estimation methods also somewhat decrease, except for the scenarios where the trait is quantitative and n=2000, and the trait is qualitative and n=500. Further, by comparing the figures for different values of τ (Figure 3, Figures S7, S9 and S11 vs. Figure 4, Figures S8, S10 and S12, respectively), it can be found that the overall widths of the HPDIs or the CIs obtained by the four interval estimation methods with τ=0.6 are generally smaller than those with τ=1, except for those with η=1. Lastly, as the trait turns from qualitative into quantitative (Figures S9–S12 vs. Figure 3, Figure 4, Figures S7 and S8, respectively) or the sample size increases (Figure 3, Figure 4, Figures S9 and S10 vs. Figures S7, S8, S11 and S12, respectively), the performances of the four interval estimation methods are greatly improved.

### 3.3. Application to MCTFR Data

The MCTFR Genome-Wide Association Study of Behavioral Disinhibition is a family-based epidemiological study of substance abuse and related psychopathology. The dataset can be made available from the database of Genotypes and Phenotypes with accession numbers 86747-6 and 95621-5 (https://www.ncbi.nlm.nih.gov/projects/gap/cgi-bin/study.cgi?study_id=phs000620.v1.p1, accessed on 5 January 2022). The dataset includes 2183 families and 7377 participants (3831 female subjects and 3546 male subjects). Among them, only 5960 subjects have both the phenotypic and genotypic data, while others do not have phenotypic data or do not have genotypic data. There are five quantitative traits included in this dataset: the nicotine composite score, the alcohol consumption composite score, the alcohol dependence composite score, the illicit drug composite score and the non-substance use related behavioral disinhibition composite score. To avoid the influence of family structure on the results, we exclude offspring from the real data application. At the same time, we only need the information of female subjects, so we also exclude male subjects from the analysis. Meanwhile, 12,354 SNPs on the X chromosome are included in the dataset. We use the following quality control criteria to filter the SNPs [48,49]: (1) genotype call rate being less than 99%, (2) MAF being smaller than $1\times 10^{-5}$, (3) individual call rate being below 99%, and (4) the *p* value of the Hardy–Weinberg equilibrium test being less than $1\times 10^{-6}$. Finally, 1994 female subjects and 12,342 SNPs on the X chromosome are utilized to conduct real data analysis. Since we estimate the degree of the skewness of the XCI based on genes, we first need to find the genes which each SNP belongs to. Based on the GRCH38 (Genome Reference Consortium Human Genome Build 38, https://uswest.ensembl.org/, accessed on 25 February 2022) reference, we use the “getBM” function in the R package “biomaRt” to match the SNPs to the genes on the X chromosome [45]. As such, we find 733 matched genes, while there are some genes containing only a single SNP in the dataset. As there have been several methods available to estimate the degree of the skewness of the XCI for a single SNP, we exclude genes consisting of only one SNP. Therefore, only 493 genes are included in the subsequent analysis.

Note that estimating γ requires the genes on the X chromosome to be associated with the traits. So, we need to test if the associations between the genes and the traits exist before using our proposed methods to estimate the degree of skewness of the XCI. Notice that the five traits in the MCTFR dataset do not follow normal distributions; therefore, we use the rank-based inverse normal transformation to transform the trait data [50]. Further, to adjust the effects of other variables, we incorporate two covariates, age and year of birth, into the application [48]. Due to the fact that we only use female subjects, we still apply the adaptive sum test proposed by Iuliana et al. [35] to test for the association between each gene and each trait. Unlike other multi-locus association analysis methods, when there are both rare and common variants in a gene, the adaptive sum test still maintains high test power. We set the significance level to be $\alpha =0.05/\left(5\times 493\right)=2.03\times 10^{-5}$ based on the Bonferroni correction. After identifying the genes associated with the traits, we calculate the four point estimates of γ (${\hat{\gamma }}_{GBN}$, ${\hat{\gamma }}_{GBU}$, ${\hat{\gamma }}_{GPF}$ and ${\hat{\gamma }}_{GF}$), and then use the GBN, GBU, PF and Fieller’s methods to obtain the corresponding HPDIs or CIs.

We finally identify only one gene, *TMEM47*, statistically significantly associated with the alcohol dependence composite score (*p* value $=2.32\times 10^{-6}$). There are two SNPs (*rs10522027* and *rs5928615*) included in the gene. The estimated MAFs of these two SNPs are 0.1407 and 0.0998, respectively, which means that both SNPs only contain common variants. It has been confirmed that *TMEM47* is located in the NC_000023.11 region and includes three exons. Studies have shown that the gene is expressed in the bladder, adipose and 23 other tissues and found that the overexpression of *TMEM47* may induce resistance in patients to certain chemotherapy drugs [51,52]. The four point estimates (${\hat{\gamma }}_{GBN}$, ${\hat{\gamma }}_{GBU}$, ${\hat{\gamma }}_{GPF}$ and ${\hat{\gamma }}_{GF}$) of γ for the gene are 0.4703, 0.4547, 0.4816 and 0.4847, and the 95% HPDIs or CIs derived by the GBN, GBU, PF and Fieller’s methods are (0.0023, 1.2380), (0.0337, 1.3083), (0.0562, 1.2410) and (0.0557, 1.3896), respectively. That is to say, the point estimates are all less than 0.5, while the 95% HPDIs or CIs all contain 1, which means that the XCI pattern for *TMEM47* on the alcohol dependence composite score may be the XCI-R or the XCI-E. By comparing the interval widths of these four interval estimation methods, we find that the width of the CI obtained by the PF method is the shortest, followed by the HPDI obtained by the GBN method, and the longest is the CI yielded by the Fieller’s method.

## 4. Discussion

In this paper, we propose four point estimates (${\hat{\gamma }}_{GBN}$, ${\hat{\gamma }}_{GBU}$, ${\hat{\gamma }}_{GPF}$ and ${\hat{\gamma }}_{GF}$) and four interval estimation methods (GBN, GBU, PF and Fieller) of the degree of the skewness of the XCI for a gene (i.e., γ). Among the point estimates, ${\hat{\gamma }}_{GF}$ is constructed by truncating the ratio of the two regression coefficients by the interval [0, 2]. And, ${\hat{\gamma }}_{GPF}$ is obtained by choosing the penalty parameter $\lambda =Z_{1-\alpha /2}^{2}/4$, and respectively correcting the denominator and the numerator, which is also truncated by [0, 2]. Both the ${\hat{\gamma }}_{GBN}$ and ${\hat{\gamma }}_{GBU}$ are developed, based on the Bayesian theory, by considering the prior information of γ∈[0, 2], and the corresponding prior distributions of γ are respectively a truncated normal distribution and a uniform distribution. Use of$\;\;{\hat{\gamma }}_{GBN}$ and ${\hat{\gamma }}_{GBU}$ can avoid the extreme point estimates of γ (0 or 2) occurring. Among the interval estimation methods, the Fieller’s method has been widely used to construct the CIs of a ratio estimate. The PF method can always get the bounded CIs by choosing an appropriate penalty parameter. The GBN and GBU methods calculate the HPDIs of the samples randomly chosen from the approximate posterior distributions of γ as the credible intervals, which can avoid empty sets, noninformative intervals (i.e., [0, 2]) and discontinuous intervals to occur. We conducted extensive simulation studies to compare their performances, by simulating different types of traits (quantitative and qualitative), different sample sizes (n=500 and 2000), different proportions of rare variants among all the SNPs considered (η=0, 0.4 and 1), and different proportions of the SNPs with positive effects among all the SNPs considered (τ=0.6 and 1). The simulation results showed that there may exist some extreme point estimates for ${\hat{\gamma }}_{GPF}$ and ${\hat{\gamma }}_{GF}$, especially when the sample size is small or the proportion of rare variants is high. The least MSE, in most situations, is derived from ${\hat{\gamma }}_{GBN}$, and the MSEs of ${\hat{\gamma }}_{GBN}$ and ${\hat{\gamma }}_{GBU}$ are smaller than those of ${\hat{\gamma }}_{GPF}$ and ${\hat{\gamma }}_{GF}$. As for the interval estimation, the CIs derived by the Fieller’s method may be empty sets, noninformative intervals and discontinuous intervals. Although the PF method can avoid discontinuous intervals, the resulting CIs can be empty sets and noninformative intervals. In addition, most of the CPs of the GBN and GBU methods can be controlled around 95%, and a larger sample size is required only when the trait is qualitative and all the SNPs are rare variants. For qualitative traits, the CPs of the PF method appear a little low when the sample size is relatively small. However, the CPs of the Fieller’s method seem to be well controlled, which is due to the large proportion of noninformative intervals. The GBN method has the smallest $W_{mean}$, $W_{median}$ and $W_{iqr}$ in most situations, and the least $W_{sd}$ under all the simulation settings. Therefore, we recommend using ${\hat{\gamma }}_{GBN}$ and the GBN method to estimate the degree of the XCI skewing in practical applications.

On the other hand, concerning the simulation settings and the simulation results, we further discuss the following issues. Firstly, we consider the influence of the proportion of rare variants (η) and the proportion of the SNPs with positive effects (τ) among all the SNPs in the gene under study on the estimation results. When τ=0.6 and other parameters are fixed, the proportions of the extreme values (0 and 2) for ${\hat{\gamma }}_{GPF}$ and ${\hat{\gamma }}_{GF}$ with η=0 are generally less than those with η=1, while they have no obvious trends for other cases of different values of η and τ. In general, the MSEs of the four point estimates generally become larger as η changes from 0, 0.4 to 1 and other parameters are kept unchanged. The four point estimates with τ=0.6 always have smaller MSEs than τ=1. The changing trends of the EPs, NPs and DPs of the PF and Fieller’s methods with the increase of η are related to τ. Furthermore, the EPs and NPs of the PF and Fieller’s methods with τ=0.6 generally are smaller than τ=1, while the DPs of the Fieller’s method with τ=0.6 are larger than or equal to those with τ=1. On the other hand, in the case of τ=0.6, the four interval estimation methods have larger $W_{mean}$’s and $W_{median}$’s in most cases with η changing from 0, 0.4 to 1, while for the scenario of τ=1, those of the four methods show a trend of first increasing and then decreasing on most occasions. The changing tendencies of the $W_{sd}$’s and $W_{iqr}$’s of the four methods, with η increasing, are affected by the trait type, n and τ. When other parameters are kept unchanged, the $W_{mean}$’s and $W_{median}$’s of the four methods with τ=0.6 are smaller than those with τ=1 in most cases. Besides this, the findings, by comparing the $W_{sd}$’s and $W_{iqr}$’s of the GBN, GBU and Fieller’s methods for τ=0.6 with those for τ=1, are related to the trait type and n, while the $W_{sd}$’s and $W_{iqr}$’s of the PF method with τ=0.6 are generally smaller than those with τ=1. Secondly, to better evaluate the performances of the proposed methods, we set the degrees of the XCI skewing at all the SNPs in the gene to be the same in our simulation studies. For example, when we calculate the MSEs of the point estimates and the CPs of the HPDIs or the CIs, a single true value of γ for each replicate is required. However, note that there may be different degrees of the XCI skewing at different SNPs, and, actually, we can also consider this issue in our simulation studies, although we have no appropriate evaluation indexes to assess the performances of the proposed methods for this situation. Finally, when we simulate quantitative traits, the random error $\varepsilon_{i}$ is generated from the standard normal distribution, where the standard deviation (σ) is equal to 1. To further illustrate the effect of different values of σ on the estimation results, we conducted additional simulation studies with n=2000 and assume that $\varepsilon_{i}$ follows N(0, 4), where σ=2. The corresponding results are presented in Tables S2–S4 and Figures S13–S16. As can be seen from these tables and figures, the Bayesian methods still have obvious advantages in both the point estimation and the interval estimation. Further, the four point estimation methods, and the four interval estimation methods with σ=2, perform worse than σ=1.

We applied the proposed methods to the MCTFR data and identified a gene, *TMEM47*, which is statistically significantly associated with the alcohol dependence composite score. However, although the four point estimates of γ for the gene *TMEM47* on the alcohol dependence composite score are all smaller than 0.5, the corresponding 95% HPDIs or CIs all contain 1, which means that the XCI pattern for this gene may not be the XCI-S. Further, we observed that the width of the CI obtained by the PF method is the shortest, followed by the HPDI obtained by the GBN method, and the longest was the CI yielded by the Fieller’s method. However, it should be noted that the PF method may not control the CP well (e.g., Table S3).

Last, but not least, there are still some issues in our proposed methods which need to be discussed. Firstly, we would like to further discuss the effect of the truncation by the interval [0, 2] on the point estimation and the interval estimation of γ. When we use the ${\hat{\gamma }}_{GPF}$ and ${\hat{\gamma }}_{GF}$ to estimate γ, both of them are truncated by [0, 2]. If the point estimates before the truncation (${\hat{\gamma }}^{*}$ and $\hat{\gamma }$) lie outside [0, 2], ${\hat{\gamma }}_{GPF}$ and ${\hat{\gamma }}_{GF}$ become the extreme values (0 or 2). Correspondingly, when using the PF and Fieller’s methods to construct the CIs of γ, it is easy to obtain empty sets or noninformative intervals. On the contrary, the Bayesian methods can avoid extreme point estimates, empty sets and noninformative intervals by specifying the appropriate prior distributions of γ and making full use of the constraint condition of γ∈[0, 2]. In addition, the extreme point estimate of 0 (2) means that the XCI is completely skewed towards the minor alleles (major alleles) at all the SNPs in a gene. However, these phenomena are not common in practice [2]. Meanwhile, it should be noted that empty sets and noninformative intervals are not informative, and the discontinuous CIs are also not useful, because the discontinuous CIs cannot be clearly explained in practice. Secondly, since the XCI patterns at different SNPs may be different, our estimated $\hat{\gamma }$ is just the mean degree of the skewness of the XCI over all the SNPs in the gene under study, and we cannot obtain the degree of the skewness of the XCI for each SNP in this gene. Meanwhile, in the process of estimating γ, the target allele is the minor one at each SNP, and it is not possible to distinguish the disease allele from the normal allele at each SNP. Therefore, we can only identify whether or not the XCI of the gene is skewed towards the minor alleles, but it is not possible to know whether the XCI is skewed towards the disease alleles or the normal alleles. Thirdly, the proposed Bayesian methods need to specify the prior distributions of all the unknown parameters in advance, and the selection of the prior distributions may have a certain impact on the results. For simplicity, we only considered two possible prior distributions for γ, and one prior distribution for each of the other unknown parameters. However, the prior distributions of these parameters are usually unknown, and we cannot guarantee that the weak prior distributions we used are the most appropriate. We provide an R package named GEXCIS, which is publicly available at https://github.com/Meng-KaiLi/GEXCIS (accessed on 30 April 2022), and can be used to estimate the degree of the skewness of the XCI for genes through the proposed methods in this paper. This R package also allows researchers to specify the prior distribution of each unknown parameter from their own research backgrounds. Fourthly, the Bayesian methods use the HMC algorithm for the sampling, which is not affected by the correlation between unknown parameters. Therefore, to improve computational efficiency, we assumed that all the unknown parameters are independent. However, the Bayesian methods, taking the correlation between the parameters into account, should have better performance, which is our future work. Fifthly, if the HPDIs or CIs we get contain 1, which means that the XCI pattern for the gene is the XCI-R or the XCI-E, our proposed methods cannot distinguish them. Therefore, in our future study, we will consider including males’ information to distinguish the XCI-R from the XCI-E. Finally, the proposed methods are only applicable to independent female subjects, and we will extend them in future so that they could accommodate the family data.

## 5. Conclusions

We propose four point estimates and four interval estimation methods to estimate γ of genes. Among the four point estimates, ${\hat{\gamma }}_{GF}$ may have the extreme point estimates, and ${\hat{\gamma }}_{GPF}$ can only reduce the occurrence of the extreme point estimates equal to 2, while ${\hat{\gamma }}_{GBN}$ and ${\hat{\gamma }}_{GBU}$ can avoid the extreme point estimates occurring. As for the four interval estimation methods, the Fieller’s method may derive empty sets, discontinuous intervals and noninformative intervals, and the PF method can avoid the occurrence of discontinuous intervals and get less noninformative intervals, while the GBN and GBU methods do not yield these three types of the intervals. However, it should be noted that through these proposed methods, we cannot obtain the degree of the skewness of the XCI for each SNP in the gene, and cannot know whether the XCI is skewed towards the disease alleles or the normal alleles. In summary, the point estimates obtained by the GBN method always have the least MSE, and the HPDIs of the GBN method generally have the shortest width and the lowest variation, so we recommend using the GBN method in practical applications.

## Figures and Tables

**Table 1:** Proportions (%) of extreme values of ${\hat{\gamma }}_{GPF}$ and ${\hat{\gamma }}_{GF}$ among 500 replications.

Trait	n	η ^a^	τ ^b^	${\hat{\gamma }}_{GPF}$			${\hat{\gamma }}_{GF}$		
				0	2	Total	0	2	Total
Quantitative	500	0	0.6	8.6	10.6	19.2	8.6	11.8	20.4
	500	0	1	7.6	19.2	26.8	7.6	21.4	29.0
	500	0.4	0.6	9.6	8.2	17.8	9.6	10.6	20.2
	500	0.4	1	11.2	16.0	27.2	11.2	21.2	32.4
	500	1	0.6	13.4	11.8	25.2	13.4	15.0	28.4
	500	1	1	9.0	9.0	18.0	9.0	15.8	24.8
	2000	0	0.6	5.2	6.0	11.2	5.2	6.2	11.4
	2000	0	1	5.0	9.4	14.4	5.0	9.6	14.6
	2000	0.4	0.6	5.6	4.6	10.2	5.6	5.0	10.6
	2000	0.4	1	6.4	10.8	17.2	6.4	11.2	17.6
	2000	1	0.6	9.8	7.0	16.8	9.8	7.2	17.0
	2000	1	1	1.4	12.2	13.6	1.4	13.8	15.2
Qualitative	500	0	0.6	19.6	12.8	32.4	19.6	20.0	39.6
	500	0	1	23.8	17.0	40.8	23.8	20.4	44.2
	500	0.4	0.6	18.8	12.8	31.6	18.8	22.0	40.8
	500	0.4	1	29.2	10.0	39.2	29.2	19.2	48.4
	500	1	0.6	22.0	9.0	31.0	22.0	19.2	41.2
	500	1	1	27.8	0.6	28.4	27.8	7.8	35.6
	2000	0	0.6	9.4	12.8	22.2	9.4	14.6	24.0
	2000	0	1	8.0	19.4	27.4	8.0	21.4	29.4
	2000	0.4	0.6	14.6	10.8	25.4	14.6	13.2	27.8
	2000	0.4	1	13.4	16.4	29.8	13.4	20.0	33.4
	2000	1	0.6	11.8	10.4	22.2	11.8	15.4	27.2
	2000	1	1	16.2	5.0	21.2	16.2	13.0	29.2

^a^ Proportion of rare variants among all the SNPs; ^b^ proportion of the SNPs with positive effects among all the SNPs.

**Table 2:** Mean squared errors of ${\hat{\gamma }}_{GBN}$, ${\hat{\gamma }}_{GBU}$, ${\hat{\gamma }}_{GPF}$ and ${\hat{\gamma }}_{GF}$ among 500 replications.

Trait	n	η ^a^	τ ^b^	${\hat{\gamma }}_{GBN}$	${\hat{\gamma }}_{GBU}$	${\hat{\gamma }}_{GPF}$	${\hat{\gamma }}_{GF}$
Quantitative	500	0	0.6	0.0976	0.1022	0.1236	0.1287
	500	0	1	0.1409	0.1601	0.2344	0.2549
	500	0.4	0.6	0.1335	0.1395	0.1579	0.1633
	500	0.4	1	0.1953	0.2248	0.3008	0.3601
	500	1	0.6	0.1414	0.1592	0.2079	0.2363
	500	1	1	0.1623	0.1703	0.2690	0.3475
	2000	0	0.6	0.0359	0.0379	0.0403	0.0405
	2000	0	1	0.0541	0.0642	0.0793	0.0805
	2000	0.4	0.6	0.0480	0.0512	0.0555	0.0558
	2000	0.4	1	0.0755	0.0773	0.0922	0.0959
	2000	1	0.6	0.0481	0.0509	0.0578	0.0591
	2000	1	1	0.0687	0.0727	0.0962	0.1160
Qualitative	500	0	0.6	0.2765	0.3382	0.4849	0.5503
	500	0	1	0.3100	0.4038	0.5286	0.5788
	500	0.4	0.6	0.3320	0.4087	0.5785	0.6344
	500	0.4	1	0.3826	0.4700	0.6416	0.7254
	500	1	0.6	0.3405	0.4329	0.5915	0.6369
	500	1	1	0.7519	0.7673	1.0190	1.0193
	2000	0	0.6	0.1207	0.1367	0.1595	0.1668
	2000	0	1	0.1362	0.1503	0.2133	0.2306
	2000	0.4	0.6	0.1320	0.1492	0.1937	0.2090
	2000	0.4	1	0.2168	0.2460	0.3347	0.3647
	2000	1	0.6	0.1431	0.1615	0.2144	0.2364
	2000	1	1	0.3163	0.3263	0.4684	0.5145

^a^ Proportion of rare variants among all the SNPs; ^b^ proportion of the SNPs with positive effects among all the SNPs.

**Table 3:** Proportions (%) of empty sets (EPs), noninformative intervals (NPs), and discontinuous intervals (DPs) of PF and Fieller’s methods among 500 replications.

Trait	n		η ^a^	τ ^b^	PF			Fieller		
					EP	NP	DP	EP	NP	DP
Quantitative	500	0		0.6	0.0	7.2	0.0	0.8	16.6	1.0
	500	0		1	0.0	19.0	0.0	0.2	21.8	0.0
	500	0.4		0.6	0.2	10.2	0.0	0.2	22.2	0.4
	500	0.4		1	1.4	27.2	0.0	0.4	33.8	0.0
	500	1		0.6	0.0	14.8	0.0	0.8	31.2	2.8
	500	1		1	6.8	3.6	0.0	1.0	3.6	0.0
	2000	0		0.6	0.0	0.0	0.0	0.0	0.0	0.0
	2000	0		1	0.6	0.0	0.0	0.6	0.2	0.0
	2000	0.4		0.6	0.0	0.0	0.0	0.2	0.0	0.0
	2000	0.4		1	0.0	2.4	0.0	0.4	4.2	0.0
	2000	1		0.6	0.0	0.2	0.0	0.0	2.2	0.0
	2000	1		1	0.2	0.2	0.0	0.2	0.6	0.0
Qualitative	500	0		0.6	0.0	43.4	0.0	0.6	65.0	2.8
	500	0		1	1.4	58.2	0.0	1.4	64.4	0.0
	500	0.4		0.6	0.0	45.4	0.0	0.0	68.2	4.0
	500	0.4		1	1.8	55.2	0.0	1.2	64.0	1.0
	500	1		0.6	0.0	44.0	0.0	0.4	75.0	3.6
	500	1		1	10.4	53.4	0.0	0.0	54.2	0.0
	2000	0		0.6	0.0	10.8	0.0	0.4	19.8	0.6
	2000	0		1	0.4	20.8	0.0	0.6	25.2	0.0
	2000	0.4		0.6	0.0	14.4	0.0	0.2	27.0	1.4
	2000	0.4		1	1.2	26.2	0.0	0.6	31.0	0.2
	2000	1		0.6	0.0	19.0	0.0	0.2	36.6	2.2
	2000	1		1	12.4	4.8	0.0	0.2	16.0	0.0

^a^ Proportion of rare variants among all the SNPs; ^b^ proportion of the SNPs with positive effects among all the SNPs.

**Table 4:** Coverage probability (CP, in %), $W_{mean}$ and $W_{median}$ of GBN, GBU, PF and Fieller’s methods among 500 replications.

Trait	n	η ^a^	τ ^b^	CP				$W_{mean}$				$W_{median}$			
				GBN	GBU	PF	Fieller	GBN	GBU	PF	Fieller	GBN	GBU	PF	Fieller
Quantitative	500	0	0.6	96.2	95.8	95.8	95.2	1.2357	1.2524	1.2338	1.2674	1.2439	1.2571	1.2072	1.2328
	500	0	1	96.2	97.0	97.8	95.8	1.3536	1.3695	1.4593	1.4375	1.3959	1.4152	1.4749	1.5010
	500	0.4	0.6	95.0	95.6	95.6	96.2	1.2663	1.2862	1.2815	1.3305	1.2662	1.2973	1.2449	1.2682
	500	0.4	1	95.6	96.6	94.2	95.6	1.4718	1.4977	1.5555	1.5887	1.5158	1.5571	1.6734	1.6888
	500	1	0.6	96.2	96.6	95.4	94.2	1.3457	1.3689	1.3363	1.3767	1.4001	1.4490	1.2991	1.3461
	500	1	1	94.6	95.4	87.8	93.8	1.2841	1.2983	1.2918	1.3827	1.3135	1.3316	1.4814	1.4465
	2000	0	0.6	94.6	94.2	94.8	94.6	0.7216	0.7258	0.7377	0.7413	0.7149	0.7230	0.7406	0.7425
	2000	0	1	95.8	96.0	95.8	94.2	0.8934	0.8946	0.9184	0.9249	0.9068	0.9035	0.9396	0.9469
	2000	0.4	0.6	94.0	95.4	94.4	94.6	0.7895	0.7958	0.8067	0.8152	0.7770	0.7850	0.8087	0.8124
	2000	0.4	1	95.6	96.2	97.4	96.2	1.0439	1.0505	1.0800	1.0950	1.0415	1.0420	1.0857	1.0828
	2000	1	0.6	95.8	96.6	96.2	96.2	0.8284	0.8325	0.8406	0.8539	0.7933	0.7974	0.8211	0.8190
	2000	1	1	95.4	95.6	96.6	95.0	0.9483	0.9560	0.9750	1.0066	0.9988	0.9982	1.0294	1.0527
Qualitative	500	0	0.6	92.6	94.2	95.4	95.0	1.6289	1.6667	1.6720	1.7236	1.7202	1.7749	1.8354	2.0000
	500	0	1	94.0	96.0	90.0	94.8	1.6575	1.6934	1.7053	1.7578	1.7387	1.7848	2.0000	2.0000
	500	0.4	0.6	93.0	94.6	93.6	96.0	1.6782	1.7193	1.6986	1.7668	1.7516	1.8033	1.8721	2.0000
	500	0.4	1	93.0	94.8	84.6	94.0	1.6775	1.7154	1.6108	1.7788	1.7360	1.7830	2.0000	2.0000
	500	1	0.6	92.6	94.8	93.0	96.0	1.7318	1.7742	1.6981	1.7965	1.7837	1.8283	1.8659	2.0000
	500	1	1	77.0	74.4	74.2	99.4	1.3896	1.3523	1.4088	1.8704	1.4854	1.4788	2.0000	2.0000
	2000	0	0.6	94.6	95.8	96.6	95.0	1.2519	1.2686	1.2531	1.2774	1.2388	1.2710	1.1933	1.2177
	2000	0	1	97.0	96.8	97.2	95.6	1.3832	1.4010	1.4869	1.4734	1.4162	1.4502	1.5404	1.5295
	2000	0.4	0.6	96.2	96.6	96.8	95.2	1.3468	1.3682	1.3443	1.3908	1.4163	1.4514	1.3443	1.3965
	2000	0.4	1	95.0	95.8	93.6	95.4	1.4765	1.5029	1.5565	1.5781	1.5153	1.5623	1.6985	1.6909
	2000	1	0.6	96.4	96.8	94.2	95.0	1.4216	1.4488	1.3842	1.4516	1.5241	1.5772	1.3174	1.4640
	2000	1	1	89.8	89.6	84.6	98.6	1.3833	1.3967	1.3764	1.6143	1.4576	1.4936	1.7096	1.6751

^a^ Proportion of rare variants among all the SNPs; ^b^ proportion of the SNPs with positive effects among all the SNPs.

**Table 5:** $W_{sd}$ and $W_{iqr}$ of GBN, GBU, PF and Fieller’s methods among 500 replications.

Trait	n	η ^a^	τ ^b^	$W_{sd}$				$W_{iqr}$			
				GBN	GBU	PF	Fieller	GBN	GBU	PF	Fieller
Quantitative	500	0	0.6	0.3309	0.3619	0.4066	0.4851	0.5036	0.5697	0.5403	0.6862
	500	0	1	0.3020	0.3364	0.4429	0.4948	0.4613	0.5274	0.6959	0.7625
	500	0.4	0.6	0.3312	0.3624	0.4198	0.4868	0.5334	0.5910	0.5862	0.8516
	500	0.4	1	0.2631	0.2917	0.4881	0.4498	0.3741	0.4244	0.6279	0.6390
	500	1	0.6	0.3585	0.3890	0.4492	0.5487	0.5765	0.6382	0.7346	1.0386
	500	1	1	0.2563	0.2891	0.6080	0.4568	0.3086	0.3487	0.7633	0.5616
	2000	0	0.6	0.1961	0.2118	0.2251	0.2350	0.2369	0.2684	0.2520	0.2564
	2000	0	1	0.2623	0.2874	0.3381	0.3514	0.3609	0.4000	0.4281	0.4336
	2000	0.4	0.6	0.2214	0.2419	0.2500	0.2723	0.2874	0.3203	0.2952	0.3094
	2000	0.4	1	0.3084	0.3386	0.4154	0.4447	0.3816	0.4537	0.5550	0.5927
	2000	1	0.6	0.2720	0.2941	0.3049	0.3455	0.3455	0.3840	0.3589	0.3830
	2000	1	1	0.3184	0.3442	0.4515	0.4661	0.3969	0.4519	0.6674	0.6647
Qualitative	500	0	0.6	0.2535	0.2727	0.3893	0.4565	0.2800	0.2841	0.5975	0.4816
	500	0	1	0.2005	0.2194	0.5012	0.4440	0.2140	0.2336	0.4291	0.3656
	500	0.4	0.6	0.1998	0.2129	0.3599	0.4105	0.2059	0.1966	0.5632	0.3726
	500	0.4	1	0.1611	0.1782	0.6086	0.4317	0.1748	0.1658	0.6470	0.2657
	500	1	0.6	0.1553	0.1632	0.3705	0.4144	0.1162	0.1055	0.5430	0.0354
	500	1	1	0.2933	0.3707	0.8749	0.2417	0.3847	0.5508	1.9212	0.1898
	2000	0	0.6	0.3501	0.3824	0.4415	0.5142	0.5624	0.6511	0.6639	0.8792
	2000	0	1	0.2936	0.3261	0.4417	0.4911	0.4447	0.5120	0.7372	0.8589
	2000	0.4	0.6	0.3518	0.3824	0.4411	0.5098	0.5682	0.6366	0.6747	1.0159
	2000	0.4	1	0.2487	0.2780	0.4936	0.4545	0.3529	0.3963	0.6457	0.6883
	2000	1	0.6	0.3456	0.3758	0.4350	0.5209	0.5482	0.6068	0.7529	0.9691
	2000	1	1	0.2762	0.3174	0.7095	0.3578	0.2032	0.2535	0.7992	0.3615

^a^ Proportion of rare variants among all the SNPs; ^b^ proportion of the SNPs with positive effects among all the SNPs.

## Data Availability

Publicly available datasets were analyzed in this study. This data can be found here: https://www.ncbi.nlm.nih.gov/projects/gap/cgi-bin/study.cgi?study_id=phs000620.v1.p1 (accessed on 5 January 2022).

## Supplementary Materials

The following supporting information can be downloaded at: https://www.mdpi.com/article/10.3390/genes13050827/s1, Table S1: Results of point estimations and interval estimations for γ among 500 replications with n = 3000 and 4000, η=1, τ=1 and σ=1 for qualitative trait; Table S2: MSEs of ${\hat{\gamma }}_{GBN}$, ${\hat{\gamma }}_{GBU}$, ${\hat{\gamma }}_{GPF}$ and ${\hat{\gamma }}_{GF}$ among 500 replications with n=2000 and σ=2 for quantitative trait; Table S3: CPs (%), $W_{mean}$ and $W_{median}$ of GBN, GBU, PF and Fieller’s methods among 500 replications with n=2000 and σ=2 for quantitative trait; Table S4: $W_{sd}$ and $W_{iqr}$ of GBN, GBU, PF and Fieller’s methods among 500 replications with n=2000 and σ=2 for quantitative trait; Figures S1–S6: Scatter plots of point estimates of γ against true values of γ for quantitative (σ=1) or qualitative trait with n=500 and 2000, and τ=0.6 and 1; Figures S7–S12: Widths of HPDIs or CIs of GBN, GBU, PF and Fieller’s methods against true values of γ for quantitative (σ=1) or qualitative trait with n=500 and 2000, and τ=0.6 and 1; Figures S13 and S14: Scatter plots of point estimates of γ against true values of γ for quantitative trait with n=2000, τ=0.6 and 1, and σ=2; Figures S15 and S16: Widths of HPDIs or CIs of GBN, GBU, PF and Fieller’s methods against true values of γ for quantitative trait with n=2000, τ=0.6 and 1, and σ=2.

## Appendix A

We assume that γ is the mean degree of the skewness of the XCI for the gene under study. For the ith female, we have $X_{i}=\sum_{j=1}^{J}\omega_{j}\left[\gamma g_{ij}^{\left(1\right)}+\left(2-\gamma \right)g_{ij}^{\left(2\right)}\right]$. On the other hand, when supposing that the degree of the skewness of the XCI at the jth SNP is $\gamma_{j}$, the genotypic values of genotypes $d_{j}d_{j}$, $D_{j}d_{j}$ and $D_{j}D_{j}$ at the jth SNP of the ith female are 0, $\gamma_{j}$ and 2, respectively. Similar to the construction process of $X_{i}$, we can get $X_{i}^{*}=\sum_{j=1}^{J}\omega_{j}\left[\gamma_{j}g_{ij}^{\left(1\right)}+\left(2-\gamma_{j}\right)g_{ij}^{\left(2\right)}\right]$. Under the assumption of $\sum_{i=1}^{n}X_{i}=\sum_{i=1}^{n}X_{i}^{*}$, we have

$$\sum_{i=1}^{n}\sum_{j=1}^{J}\omega_{j}\left[\gamma g_{ij}^{\left(1\right)}+\left(2-\gamma \right)g_{ij}^{\left(2\right)}\right]=\sum_{i=1}^{n}\sum_{j=1}^{J}\omega_{j}\left[\gamma_{j}g_{ij}^{\left(1\right)}+\left(2-\gamma_{j}\right)g_{ij}^{\left(2\right)}\right],$$

and

$$\gamma \sum_{i=1}^{n}\sum_{j=1}^{J}\omega_{j}\left(g_{ij}^{\left(1\right)}-g_{ij}^{\left(2\right)}\right)=\sum_{i=1}^{n}\sum_{j=1}^{J}\omega_{j}\left(g_{ij}^{\left(1\right)}-g_{ij}^{\left(2\right)}\right)\gamma_{j}.$$

Then,

$$\gamma \sum_{j=1}^{J}\omega_{j}\left(g_{.j}^{\left(1\right)}-g_{.j}^{\left(2\right)}\right)=\sum_{j=1}^{J}\omega_{j}\left(g_{.j}^{\left(1\right)}-g_{.j}^{\left(2\right)}\right)\gamma_{j},$$

where $g_{.j}^{\left(1\right)}=\sum_{i=1}^{n}g_{ij}^{\left(1\right)}$ and $g_{.j}^{\left(2\right)}=\sum_{i=1}^{n}g_{ij}^{\left(2\right)}$. Finally, we have

$$\gamma =\frac{\sum_{j=1}^{J}\omega_{j}\left(g_{.j}^{\left(1\right)}-g_{.j}^{\left(2\right)}\right)\gamma_{j}}{\sum_{j=1}^{J}\omega_{j}\left(g_{.j}^{\left(1\right)}-g_{.j}^{\left(2\right)}\right)}.$$
