# Supplementary material for: Gene-Based Methods for Estimating the Degree of the Skewness of X Chromosome Inactivation
Source: Genes (Basel). 2022 May 6;13(5):827. doi: 10.3390/genes13050827 (PMC9140558; doi:10.3390/genes13050827)
Supplement: Supplementary file 1 [file genes-13-00827-s001.zip › genes-1705332-supplementary.pdf]

Supplementary Material

Gene-Based Methods for Estimating the Degree of the Skewness of X Chromosome Inactivation

Table S1. Results of point estimations and interval estimations for γ among 500 replications with n=3000 and 4000, $\eta = 1$ and $\tau = 1$ for qualitative trait.

Estimation	Index	Method	$n = 3000$	$n = 4000$
Point estimation	Proportion of extreme values being 0 (%)	$\hat{\gamma}_{GPF}$	11.6	7.0
	Proportion of extreme values being 0 (%)	$\hat{\gamma}_{GF}$	11.6	7.0
	Proportion of extreme values being 2 (%)	$\hat{\gamma}_{GPF}$	14.6	11.0
	Proportion of extreme values being 2 (%)	$\hat{\gamma}_{GF}$	21.0	15.8
	Proportion of extreme values being 0 or 2 (%)	$\hat{\gamma}_{GPF}$	26.2	18.0
	Proportion of extreme values being 0 or 2 (%)	$\hat{\gamma}_{GF}$	32.6	22.8
	MSE	$\hat{\gamma}_{GPF}$	0.2160	0.1683
	MSE	$\hat{\gamma}_{GF}$	0.2261	0.1783
	MSE	$\hat{\gamma}_{GPF}$	0.3697	0.2515
	MSE	$\hat{\gamma}_{GF}$	0.4305	0.3061
Interval estimation	EP (%)	PF	8.2	4.8
	EP (%)	Fieller	1.4	0.0
	NP (%)	PF	1.8	1.4
	NP (%)	Fieller	5.6	3.4
	DP (%)	PF	0.0	0.0
	DP (%)	Fieller	0.2	0.0
	CP (%)	GBN	92.6	93.2
	CP (%)	GBU	93.2	94.0
	CP (%)	PF	88.2	91.4
	CP (%)	Fieller	96.4	96.4
	W_{mean}	GBN	1.3001	1.2427
	W_{mean}	GBU	1.3135	1.2557
	W_{mean}	PF	1.3283	1.2916
	W_{mean}	Fieller	1.4720	1.3757
	W_{median}	GBN	1.3539	1.3137
	W_{median}	GBU	1.3916	1.3350
	W_{median}	PF	1.6042	1.5098
	W_{median}	Fieller	1.5946	1.4778
	W_{sd}	GBN	0.2723	0.2893
	W_{sd}	GBU	0.3059	0.3206
	W_{sd}	PF	0.6399	0.5807
	W_{sd}	Fieller	0.4499	0.4627
	W_{iqr}	GBN	0.3002	0.2744
	W_{iqr}	GBU	0.3428	0.3181
	W_{iqr}	PF	0.8050	0.6589
	W_{iqr}	Fieller	0.5292	0.5173

Table S2. MSEs of $\hat{\gamma}_{GBN}$, $\hat{\gamma}_{GBU}$, $\hat{\gamma}_{GPF}$ and $\hat{\gamma}_{GF}$ among 500 replications with $n = 2000$ and $\sigma = 2$ for quantitative trait.

$\boldsymbol{\eta}$ [a]	$\boldsymbol{\tau}$ [b]	$\hat{\boldsymbol{\gamma}}_{GBN}$	$\hat{\boldsymbol{\gamma}}_{GBU}$	$\hat{\boldsymbol{\gamma}}_{GPF}$	$\hat{\boldsymbol{\gamma}}_{GF}$
0	0.6	0.1215	0.1325	0.1510	0.1607
0	1	0.1481	0.1996	0.2324	0.2500
0.4	0.6	0.1238	0.1336	0.1727	0.1829
0.4	1	0.1957	0.2196	0.3202	0.3763
1	0.6	0.1315	0.1445	0.2048	0.2212
1	1	0.1637	0.1703	0.2603	0.3208

[a] Proportion of rare variants among all the SNPs; [b] proportion of the SNPs with positive effects among all the SNPs.

Table S3. CPs (%), W_{mean} and W_{median} of GBN, GBU, PF and Fieller's methods among 500 replications with $n = 2000$ and $\sigma = 2$ for quantitative trait.

$\boldsymbol{\eta}$ [a]	$\boldsymbol{\tau}$ [b]	**CP**				$\boldsymbol{W_{mean}}$				$\boldsymbol{W_{median}}$			
		**GBN**	**GBU**	**PF**	**Fieller**	**GBN**	**GBU**	**PF**	**Fieller**	**GBN**	**GBU**	**PF**	**Fieller**
0	0.6	95.40	95.60	95.80	94.40	1.2300	1.2456	1.2557	1.2790	1.2417	1.2535	1.2283	1.2318
0	1	96.00	94.40	97.20	95.60	1.3664	1.3808	1.4696	1.4605	1.3716	1.3938	1.4829	1.4885
0.4	0.6	94.80	94.80	95.40	94.60	1.2835	1.3020	1.2819	1.3162	1.3040	1.3320	1.2313	1.2531
0.4	1	95.60	95.00	90.40	92.60	1.4447	1.4668	1.4994	1.5239	1.4833	1.5214	1.6226	1.6201
1	0.6	97.00	98.00	96.80	97.00	1.3671	1.3930	1.3895	1.4353	1.4024	1.4492	1.3481	1.3987
1	1	95.20	95.60	88.00	94.80	1.2857	1.3070	1.2886	1.4029	1.3224	1.3557	1.5152	1.4959

[a] Proportion of rare variants among all the SNPs; [b] proportion of the SNPs with positive effects among all the SNPs.

Table S4. W_{sd} and W_{iqr} of GBN, GBU, PF and Fieller's methods among 500 replications with $n = 2000$ and $\sigma = 2$ for quantitative trait.

η [a]	τ [b]	W_{sd}				W_{iqr}			
		**GBN**	**GBU**	**PF**	**Fieller**	**GBN**	**GBU**	**PF**	**Fieller**
0	0.6	0.3550	0.3884	0.4446	0.5132	0.5844	0.6631	0.6436	0.8370
0	1	0.2842	0.3186	0.4208	0.4704	0.4259	0.4848	0.6339	0.7550
0.4	0.6	0.3470	0.3801	0.4507	0.5316	0.5757	0.6536	0.6900	1.0767
0.4	1	0.2664	0.2976	0.5142	0.4707	0.3981	0.4419	0.7020	0.7523
1	0.6	0.3387	0.3686	0.4271	0.4996	0.5815	0.6371	0.7282	0.9717
1	1	0.2684	0.2977	0.6027	0.4591	0.2981	0.3539	0.7601	0.5371

[a] Proportion of rare variants among all the SNPs; [b] proportion of the SNPs with positive effects among all the SNPs.

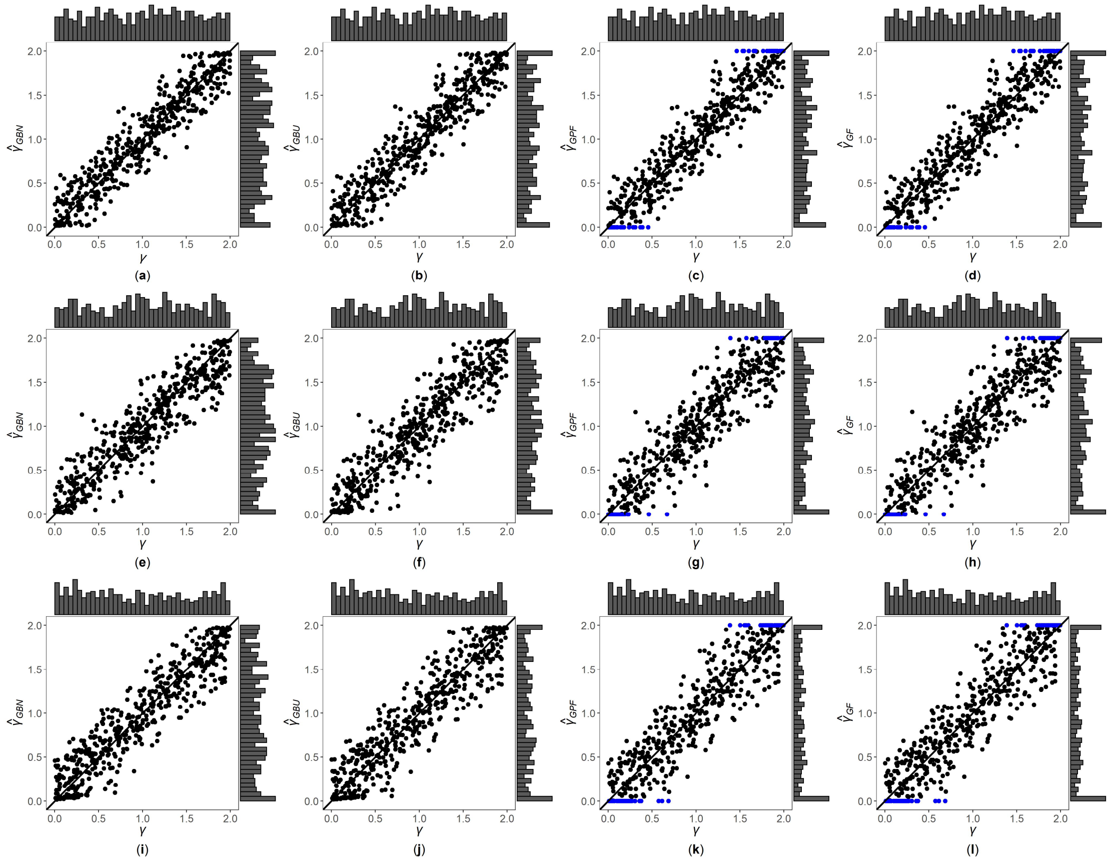

Figure S1. Scatter plots of point estimates of γ against true values of γ for quantitative trait with $n = 2000$, $\tau = 0.6$ and $\sigma = 1$. The blue points represent the extreme values (0 or 2). (**a**) $\hat{\gamma}_{GBN}$ with $\eta = 0$; (**b**) $\hat{\gamma}_{GBU}$ with $\eta = 0$; (**c**) $\hat{\gamma}_{GPF}$ with $\eta = 0$; (**d**) $\hat{\gamma}_{GF}$ with $\eta = 0$; (**e**) $\hat{\gamma}_{GBN}$ with $\eta = 0.4$; (**f**) $\hat{\gamma}_{GBU}$ with $\eta = 0.4$; (**g**) $\hat{\gamma}_{GPF}$ with $\eta = 0.4$; (**h**) $\hat{\gamma}_{GF}$ with $\eta = 0.4$; (**i**) $\hat{\gamma}_{GBN}$ with $\eta = 1$; (**j**) $\hat{\gamma}_{GBU}$ with $\eta = 1$; (**k**) $\hat{\gamma}_{GPF}$ with $\eta = 1$; (**l**) $\hat{\gamma}_{GF}$ with $\eta = 1$.

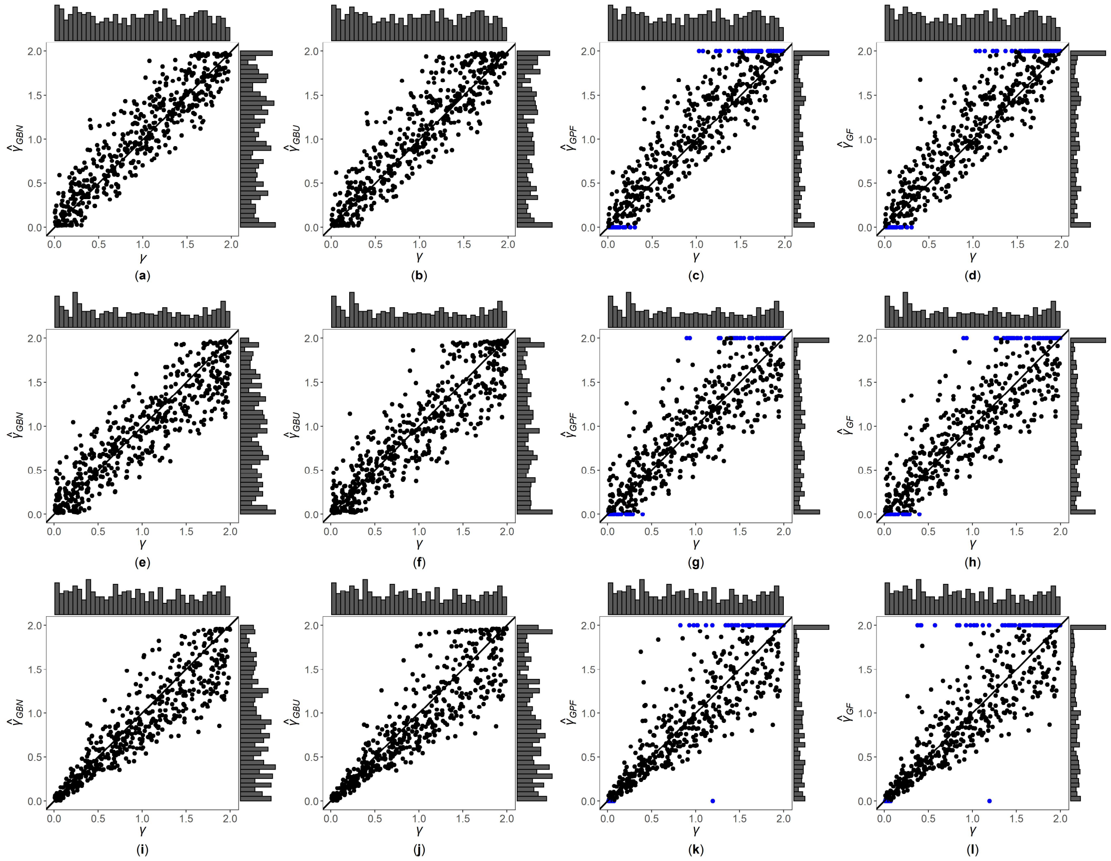

Figure S2. Scatter plots of point estimates of γ against true values of γ for quantitative trait with $n = 2000$, $\tau = 1$ and $\sigma = 1$. The blue points represent the extreme values (0 or 2). (**a**) $\hat{\gamma}_{GBN}$ with $\eta = 0$; (**b**) $\hat{\gamma}_{GBU}$ with $\eta = 0$; (**c**) $\hat{\gamma}_{GPF}$ with $\eta = 0$; (**d**) $\hat{\gamma}_{GF}$ with $\eta = 0$; (**e**) $\hat{\gamma}_{GBN}$ with $\eta = 0.4$; (**f**) $\hat{\gamma}_{GBU}$ with $\eta = 0.4$; (**g**) $\hat{\gamma}_{GPF}$ with $\eta = 0.4$; (**h**) $\hat{\gamma}_{GF}$ with $\eta = 0.4$; (**i**) $\hat{\gamma}_{GBN}$ with $\eta = 1$; (**j**) $\hat{\gamma}_{GBU}$ with $\eta = 1$; (**k**) $\hat{\gamma}_{GPF}$ with $\eta = 1$; (**l**) $\hat{\gamma}_{GF}$ with $\eta = 1$.

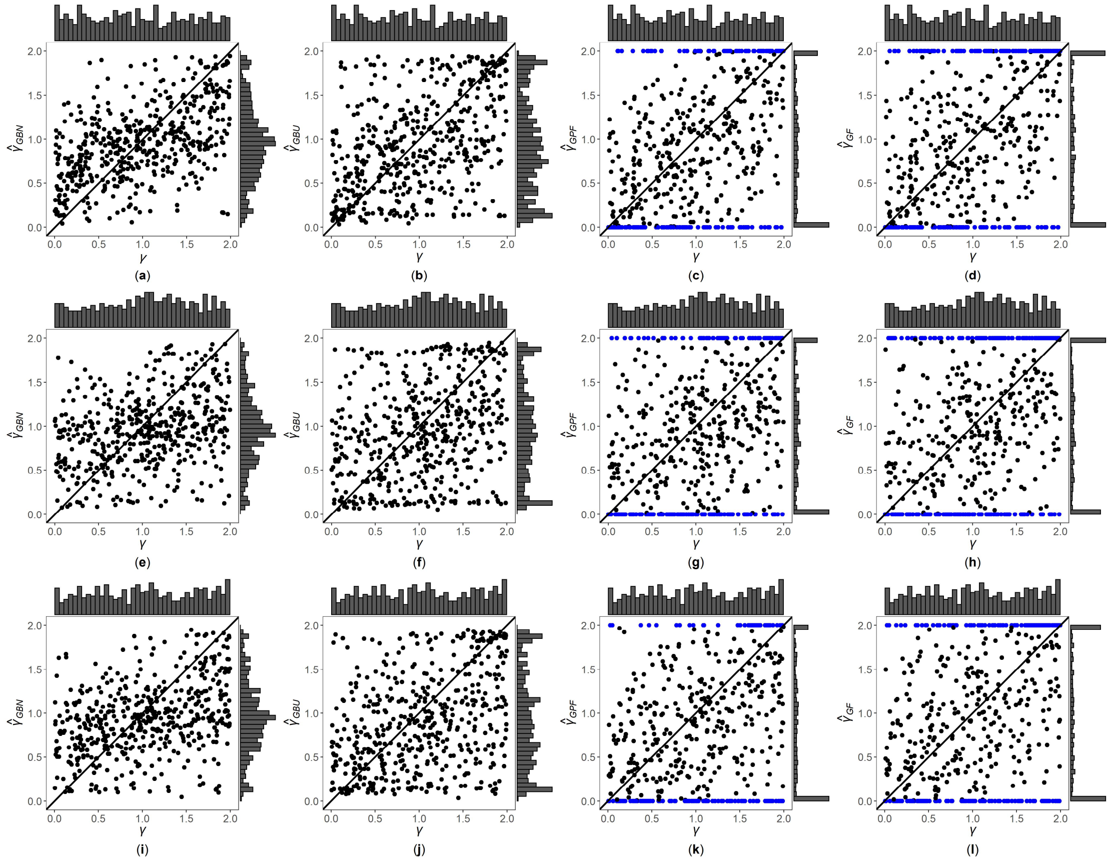

Figure S3. Scatter plots of point estimates of γ against true values of γ for qualitative trait with $n = 500$ and $\tau = 0.6$. The blue points represent the extreme values (0 or 2). (**a**) $\hat{\gamma}_{GBN}$ with $\eta = 0$; (**b**) $\hat{\gamma}_{GBU}$ with $\eta = 0$; (**c**) $\hat{\gamma}_{GPF}$ with $\eta = 0$; (**d**) $\hat{\gamma}_{GF}$ with $\eta = 0$; (**e**) $\hat{\gamma}_{GBN}$ with $\eta = 0.4$; (**f**) $\hat{\gamma}_{GBU}$ with $\eta = 0.4$; (**g**) $\hat{\gamma}_{GPF}$ with $\eta = 0.4$; (**h**) $\hat{\gamma}_{GF}$ with $\eta = 0.4$; (**i**) $\hat{\gamma}_{GBN}$ with $\eta = 1$; (**j**) $\hat{\gamma}_{GBU}$ with $\eta = 1$; (**k**) $\hat{\gamma}_{GPF}$ with $\eta = 1$; (**l**) $\hat{\gamma}_{GF}$ with $\eta = 1$.

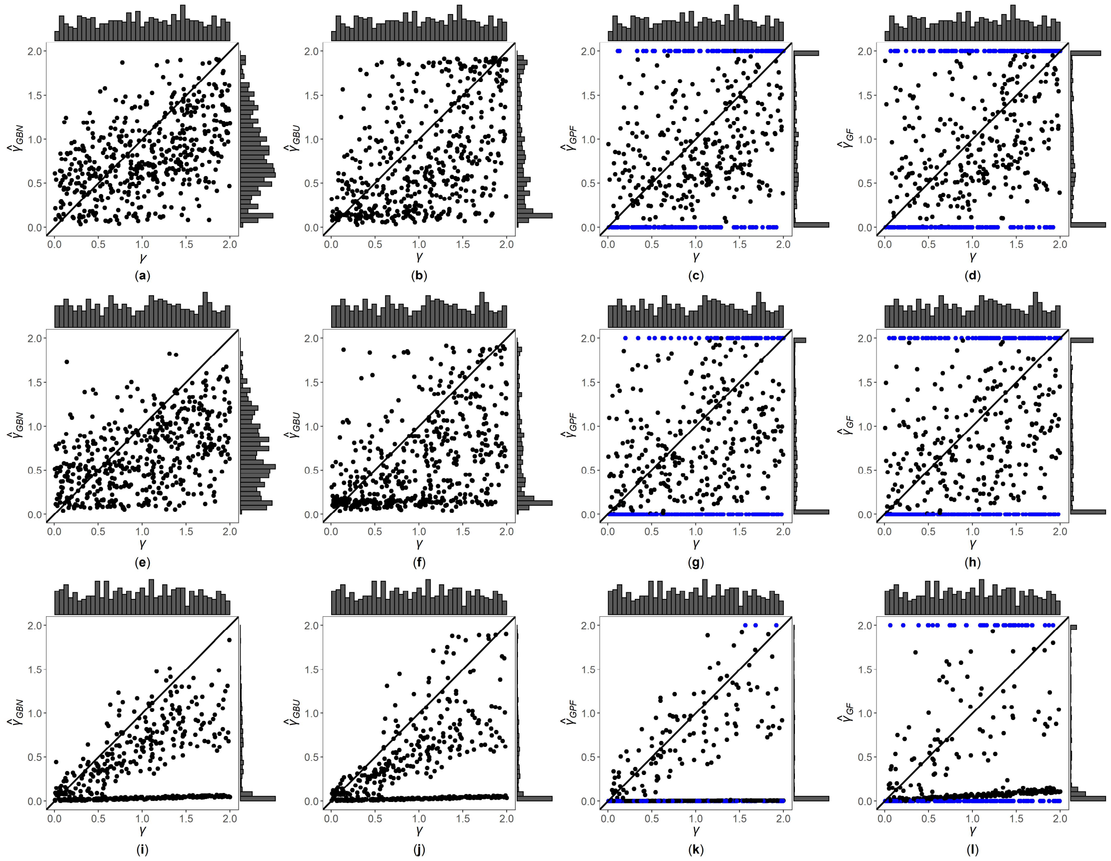

Figure S4. Scatter plots of point estimates of γ against true values of γ for qualitative trait with $n = 500$ and $\tau = 1$. The blue points represent the extreme values (0 or 2). (**a**) $\hat{\gamma}_{GBN}$ with $\eta = 0$; (**b**) $\hat{\gamma}_{GBU}$ with $\eta = 0$; (**c**) $\hat{\gamma}_{GPF}$ with $\eta = 0$; (**d**) $\hat{\gamma}_{GF}$ with $\eta = 0$; (**e**) $\hat{\gamma}_{GBN}$ with $\eta = 0.4$; (**f**) $\hat{\gamma}_{GBU}$ with $\eta = 0.4$; (**g**) $\hat{\gamma}_{GPF}$ with $\eta = 0.4$; (**h**) $\hat{\gamma}_{GF}$ with $\eta = 0.4$; (**i**) $\hat{\gamma}_{GBN}$ with $\eta = 1$; (**j**) $\hat{\gamma}_{GBU}$ with $\eta = 1$; (**k**) $\hat{\gamma}_{GPF}$ with $\eta = 1$; (**l**) $\hat{\gamma}_{GF}$ with $\eta = 1$.

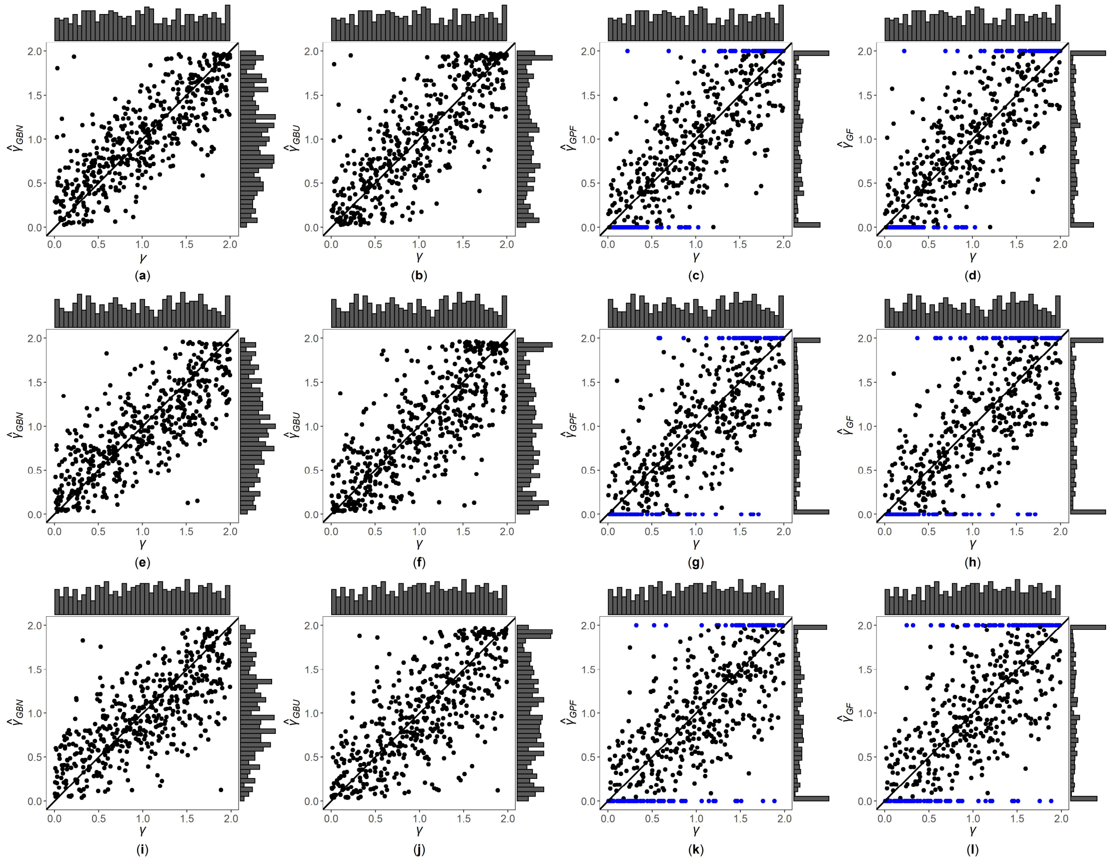

Figure S5. Scatter plots of point estimates of γ against true values of γ for qualitative trait with $n = 2000$ and $\tau = 0.6$. The blue points represent the extreme values (0 or 2). (**a**) $\hat{\gamma}_{GBN}$ with $\eta = 0$; (**b**) $\hat{\gamma}_{GBU}$ with $\eta = 0$; (**c**) $\hat{\gamma}_{GPF}$ with $\eta = 0$; (**d**) $\hat{\gamma}_{GF}$ with $\eta = 0$; (**e**) $\hat{\gamma}_{GBN}$ with $\eta = 0.4$; (**f**) $\hat{\gamma}_{GBU}$ with $\eta = 0.4$; (**g**) $\hat{\gamma}_{GPF}$ with $\eta = 0.4$; (**h**) $\hat{\gamma}_{GF}$ with $\eta = 0.4$; (**i**) $\hat{\gamma}_{GBN}$ with $\eta = 1$; (**j**) $\hat{\gamma}_{GBU}$ with $\eta = 1$; (**k**) $\hat{\gamma}_{GPF}$ with $\eta = 1$; (**l**) $\hat{\gamma}_{GF}$ with $\eta = 1$.

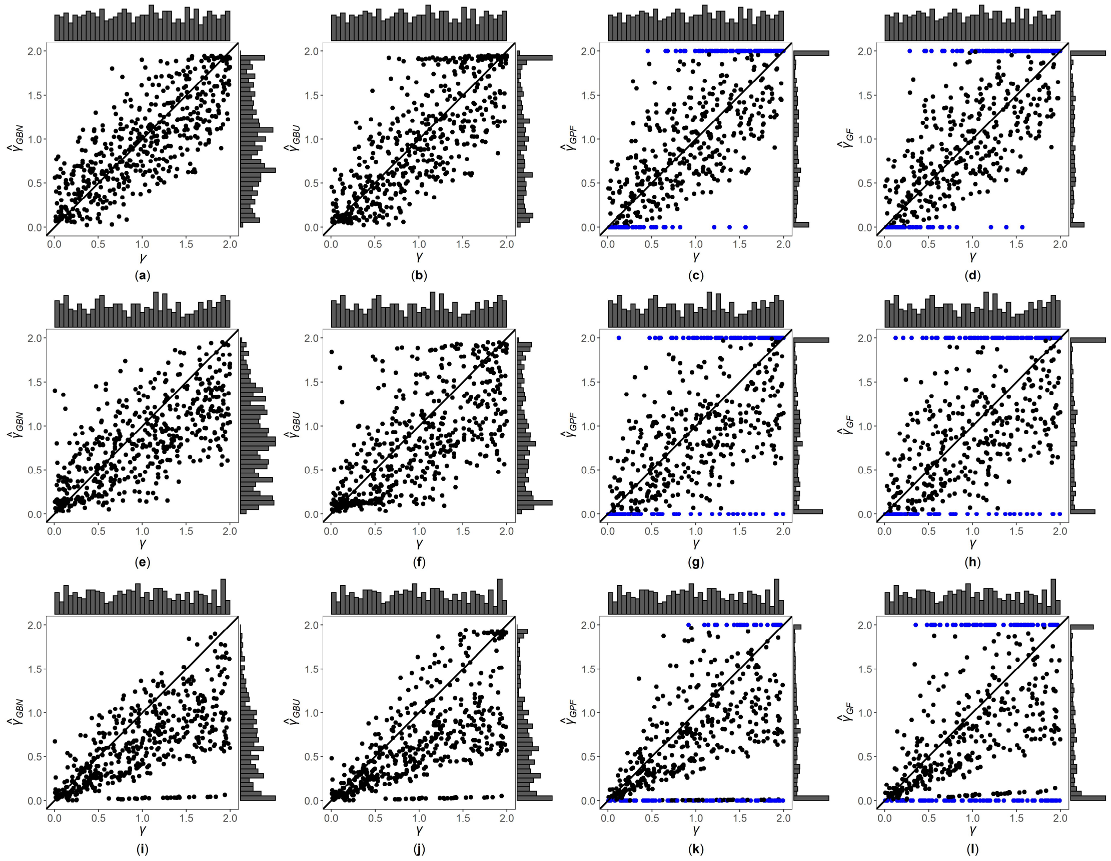

Figure S6. Scatter plots of point estimates of γ against true values of γ for qualitative trait with $n = 2000$ and $\tau = 1$. The blue points represent the extreme values (0 or 2). (**a**) $\hat{\gamma}_{GBN}$ with $\eta = 0$; (**b**) $\hat{\gamma}_{GBU}$ with $\eta = 0$; (**c**) $\hat{\gamma}_{GPF}$ with $\eta = 0$; (**d**) $\hat{\gamma}_{GF}$ with $\eta = 0$; (**e**) $\hat{\gamma}_{GBN}$ with $\eta = 0.4$; (**f**) $\hat{\gamma}_{GBU}$ with $\eta = 0.4$; (**g**) $\hat{\gamma}_{GPF}$ with $\eta = 0.4$; (**h**) $\hat{\gamma}_{GF}$ with $\eta = 0.4$; (**i**) $\hat{\gamma}_{GBN}$ with $\eta = 1$; (**j**) $\hat{\gamma}_{GBU}$ with $\eta = 1$; (**k**) $\hat{\gamma}_{GPF}$ with $\eta = 1$; (**l**) $\hat{\gamma}_{GF}$ with $\eta = 1$.

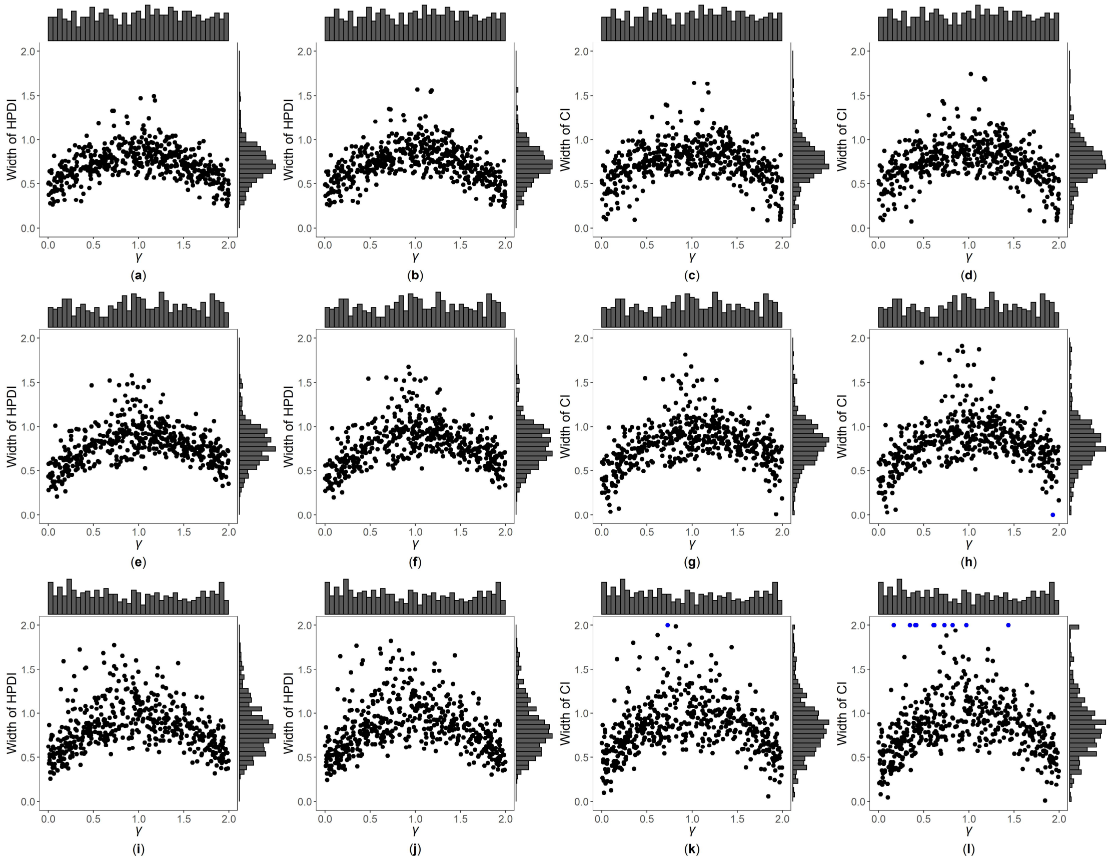

Figure S7. Widths of HPDIs or CIs of GBN, GBU, PF and Fieller's methods against true values of γ for quantitative trait with $n = 2000$, $\tau = 0.6$ and $\sigma = 1$. The blue points represent the widths of the empty sets or the noninformative intervals. (**a**) GBN with $\eta = 0$; (**b**) GBU with $\eta = 0$; (**c**) PF with $\eta = 0$; (**d**) Fieller with $\eta = 0$; (**e**) GBN with $\eta = 0.4$; (**f**) GBU with $\eta = 0.4$; (**g**) PF with $\eta = 0.4$; (**h**) Fieller with $\eta = 0.4$; (**i**) GBN with $\eta = 1$; (**j**) GBU with $\eta = 1$; (**k**) PF with $\eta = 1$; (**l**) Fieller with $\eta = 1$.

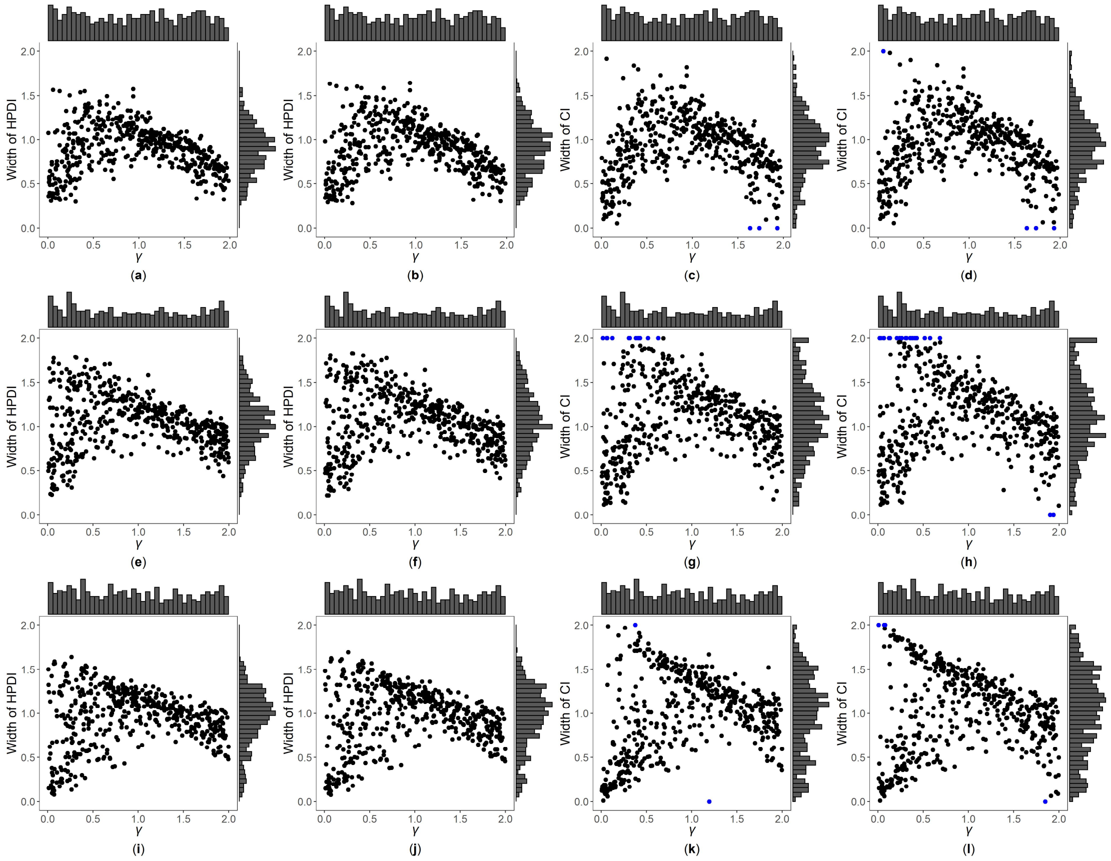

Figure S8. Widths of HPDIs or CIs of GBN, GBU, PF and Fieller's methods against true values of γ for quantitative trait with $n = 2000$, $\tau = 1$ and $\sigma = 1$. (**a**) GBN with $\eta = 0$; (**b**) GBU with $\eta = 0$; (**c**) PF with $\eta = 0$; (**d**) Fieller with $\eta = 0$; (**e**) GBN with $\eta = 0.4$; (**f**) GBU with $\eta = 0.4$; (**g**) PF with $\eta = 0.4$; (**h**) Fieller with $\eta = 0.4$; (**i**) GBN with $\eta = 1$; (**j**) GBU with $\eta = 1$; (**k**) PF with $\eta = 1$; (**l**) Fieller with $\eta = 1$.

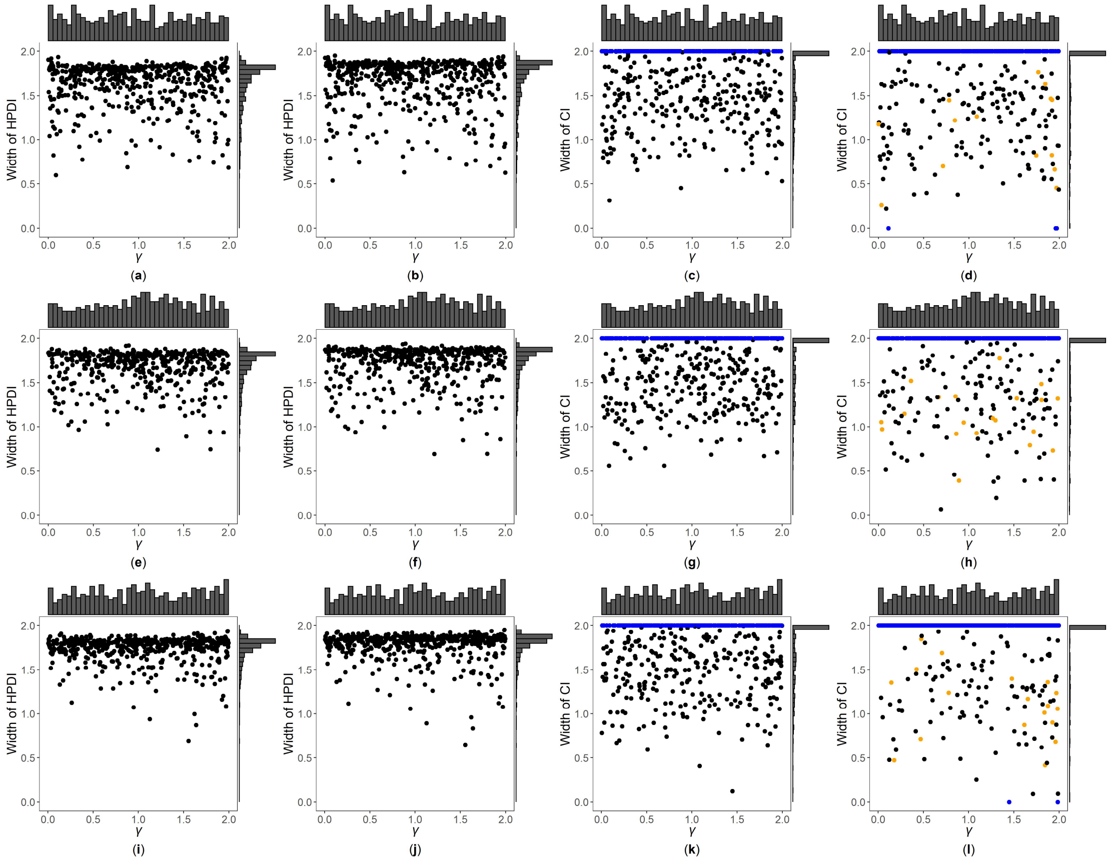

Figure S9. Widths of HPDIs or CIs of GBN, GBU, PF and Fieller's methods against true values of γ for qualitative trait with $n = 500$ and $\tau = 0.6$. The blue points represent the widths of the empty sets or the noninformative intervals, and the orange points represent the widths of the discontinuous intervals. (**a**) GBN with $\eta = 0$; (**b**) GBU with $\eta = 0$; (**c**) PF with $\eta = 0$; (**d**) Fieller with $\eta = 0$; (**e**) GBN with $\eta = 0.4$; (**f**) GBU with $\eta = 0.4$; (**g**) PF with $\eta = 0.4$; (**h**) Fieller with $\eta = 0.4$; (**i**) GBN with $\eta = 1$; (**j**) GBU with $\eta = 1$; (**k**) PF with $\eta = 1$; (**l**) Fieller with $\eta = 1$.

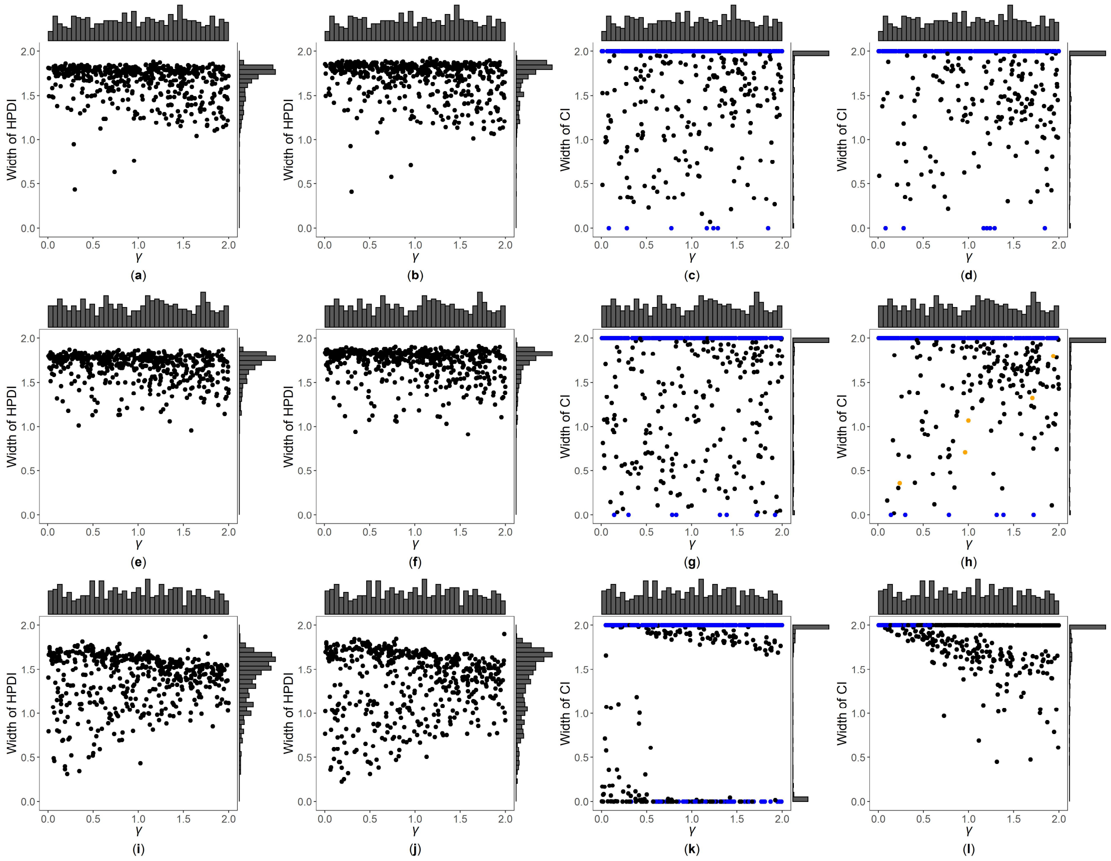

Figure S10. Widths of HPDIs or CIs of GBN, GBU, PF and Fieller's methods against true values of γ for qualitative trait with $n = 500$ and $\tau = 1$. The blue points represent the widths of the empty sets or the noninformative intervals, and the orange points represent the widths of the discontinuous intervals. (**a**) GBN with $\eta = 0$; (**b**) GBU with $\eta = 0$; (**c**) PF with $\eta = 0$; (**d**) Fieller with $\eta = 0$; (**e**) GBN with $\eta = 0.4$; (**f**) GBU with $\eta = 0.4$; (**g**) PF with $\eta = 0.4$; (**h**) Fieller with $\eta = 0.4$; (**i**) GBN with $\eta = 1$; (**j**) GBU with $\eta = 1$; (**k**) PF with $\eta = 1$; (**l**) Fieller with $\eta = 1$.

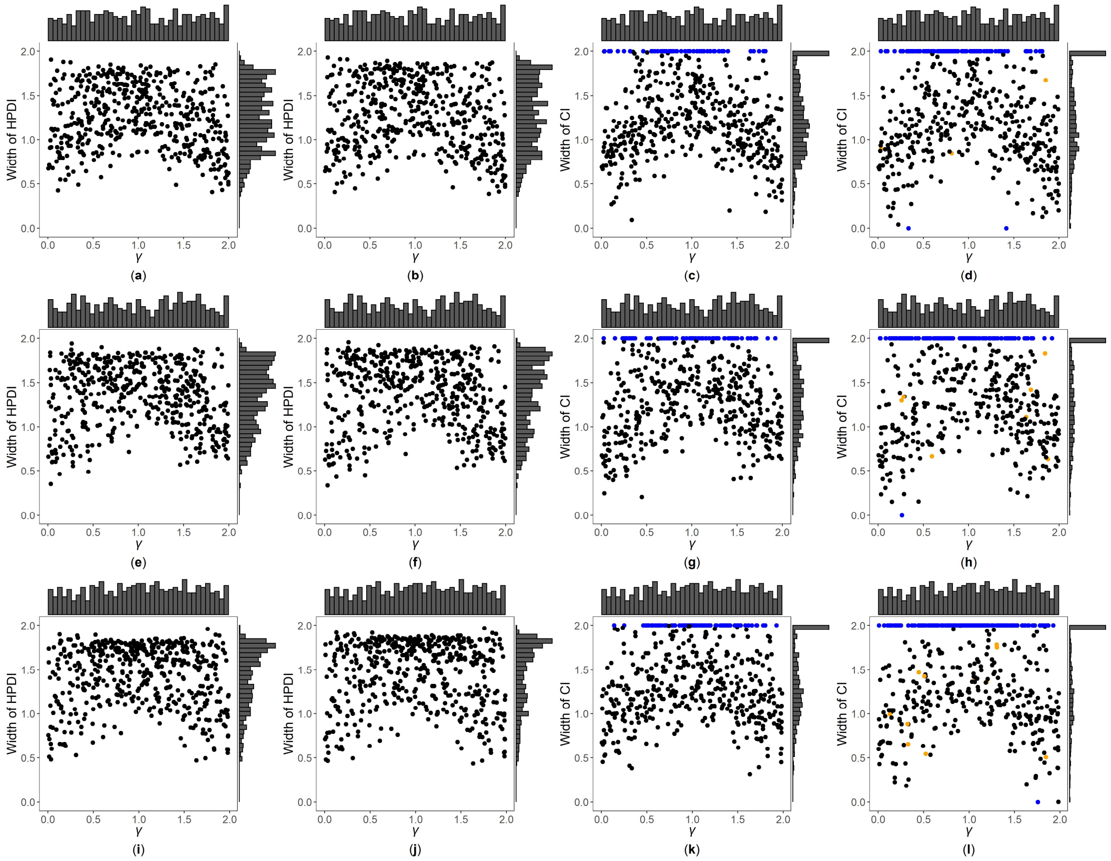

Figure S11. Widths of HPDIs or CIs of GBN, GBU, PF and Fieller's methods against true values of γ for qualitative trait with $n = 2000$ and $\tau = 0.6$. The blue points represent the widths of the empty sets or the noninformative intervals, and the orange points represent the widths of the discontinuous intervals. (**a**) GBN with $\eta = 0$; (**b**) GBU with $\eta = 0$; (**c**) PF with $\eta = 0$; (**d**) Fieller with $\eta = 0$; (**e**) GBN with $\eta = 0.4$; (**f**) GBU with $\eta = 0.4$; (**g**) PF with $\eta = 0.4$; (**h**) Fieller with $\eta = 0.4$; (**i**) GBN with $\eta = 1$; (**j**) GBU with $\eta = 1$; (**k**) PF with $\eta = 1$; (**l**) Fieller with $\eta = 1$.

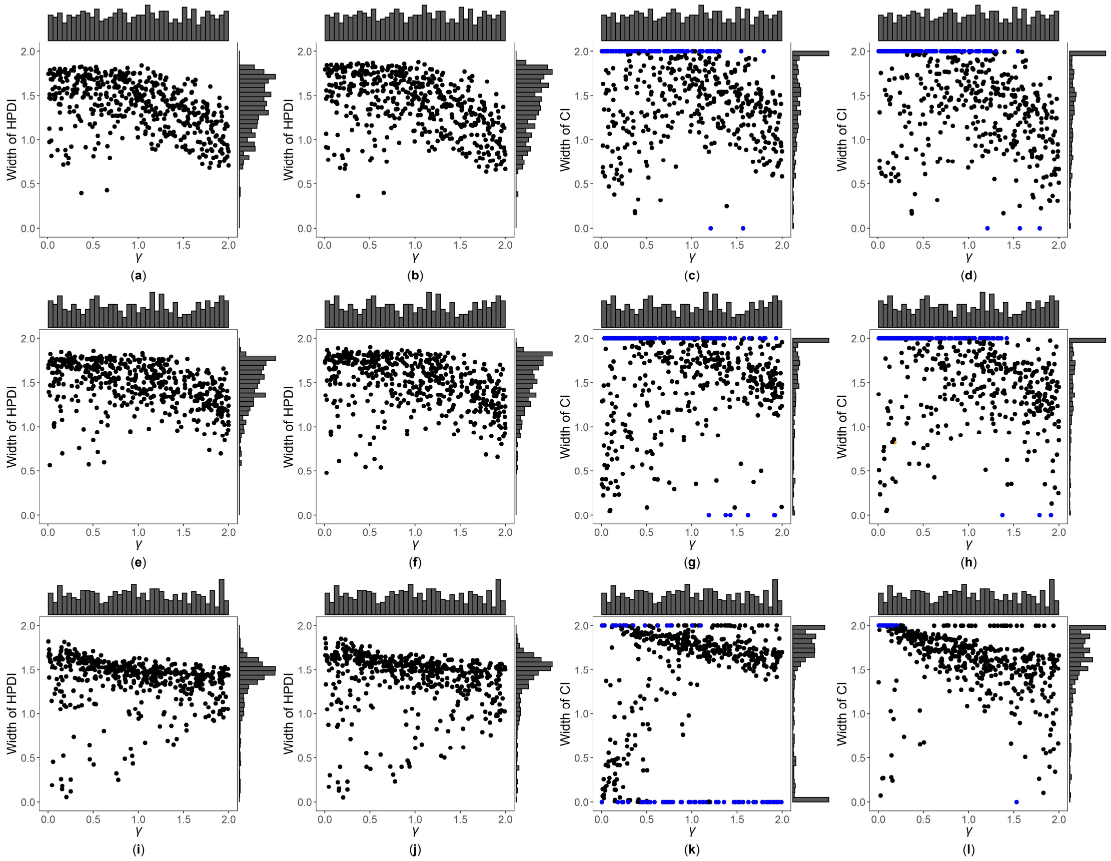

Figure S12. Widths of HPDIs or CIs of GBN, GBU, PF and Fieller's methods against true values of γ for qualitative trait with $n = 2000$ and $\tau = 1$. The blue points represent the widths of the empty sets or the noninformative intervals, and the orange points represent the widths of the discontinuous intervals. (**a**) GBN with $\eta = 0$; (**b**) GBU with $\eta = 0$; (**c**) PF with $\eta = 0$; (**d**) Fieller with $\eta = 0$; (**e**) GBN with $\eta = 0.4$; (**f**) GBU with $\eta = 0.4$; (**g**) PF with $\eta = 0.4$; (**h**) Fieller with $\eta = 0.4$; (**i**) GBN with $\eta = 1$; (**j**) GBU with $\eta = 1$; (**k**) PF with $\eta = 1$; (**l**) Fieller with $\eta = 1$.

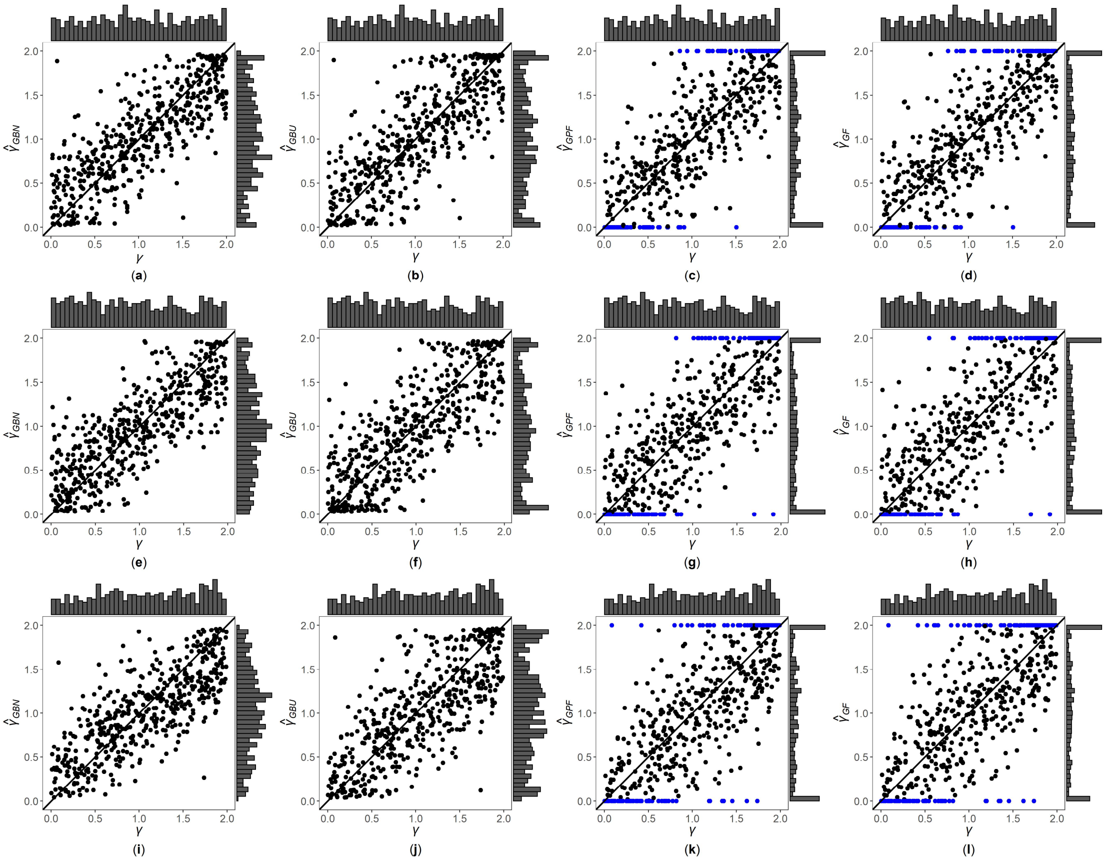

Figure S13. Scatter plots of point estimates of γ against true values of γ for quantitative trait with $n = 2000$, $\tau = 0.6$ and $\sigma = 2$. The blue points represent the extreme values (0 or 2). (**a**) $\hat{\gamma}_{GBN}$ with $\eta = 0$; (**b**) $\hat{\gamma}_{GBU}$ with $\eta = 0$; (**c**) $\hat{\gamma}_{GPF}$ with $\eta = 0$; (**d**) $\hat{\gamma}_{GF}$ with $\eta = 0$; (**e**) $\hat{\gamma}_{GBN}$ with $\eta = 0.4$; (**f**) $\hat{\gamma}_{GBU}$ with $\eta = 0.4$; (**g**) $\hat{\gamma}_{GPF}$ with $\eta = 0.4$; (**h**) $\hat{\gamma}_{GF}$ with $\eta = 0.4$; (**i**) $\hat{\gamma}_{GBN}$ with $\eta = 1$; (**j**) $\hat{\gamma}_{GBU}$ with $\eta = 1$; (**k**) $\hat{\gamma}_{GPF}$ with $\eta = 1$; (**l**) $\hat{\gamma}_{GF}$ with $\eta = 1$.

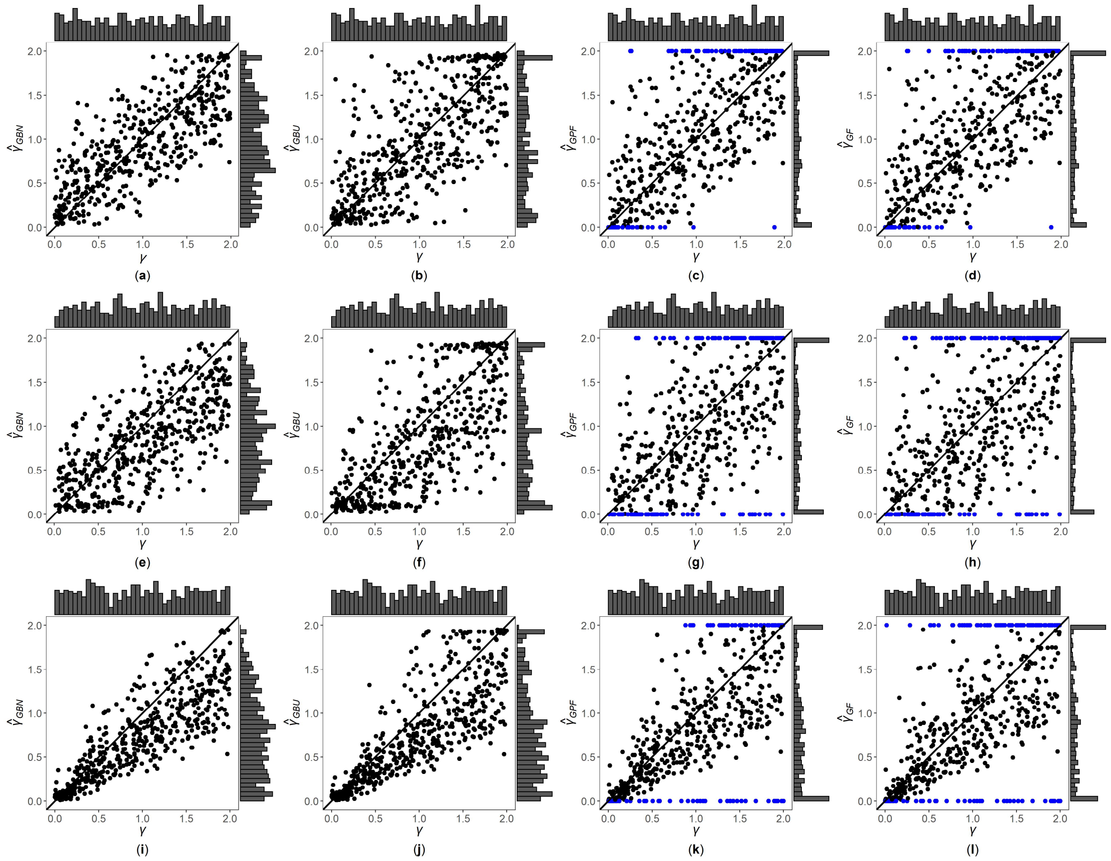

Figure S14. Scatter plots of point estimates of γ against true values of γ for quantitative trait with $n = 2000$, $\tau = 1$ and $\sigma = 2$. The blue points represent the extreme values (0 or 2). (**a**) $\hat{\gamma}_{GBN}$ with $\eta = 0$; (**b**) $\hat{\gamma}_{GBU}$ with $\eta = 0$; (**c**) $\hat{\gamma}_{GPF}$ with $\eta = 0$; (**d**) $\hat{\gamma}_{GF}$ with $\eta = 0$; (**e**) $\hat{\gamma}_{GBN}$ with $\eta = 0.4$; (**f**) $\hat{\gamma}_{GBU}$ with $\eta = 0.4$; (**g**) $\hat{\gamma}_{GPF}$ with $\eta = 0.4$; (**h**) $\hat{\gamma}_{GF}$ with $\eta = 0.4$; (**i**) $\hat{\gamma}_{GBN}$ with $\eta = 1$; (**j**) $\hat{\gamma}_{GBU}$ with $\eta = 1$; (**k**) $\hat{\gamma}_{GPF}$ with $\eta = 1$; (**l**) $\hat{\gamma}_{GF}$ with $\eta = 1$.

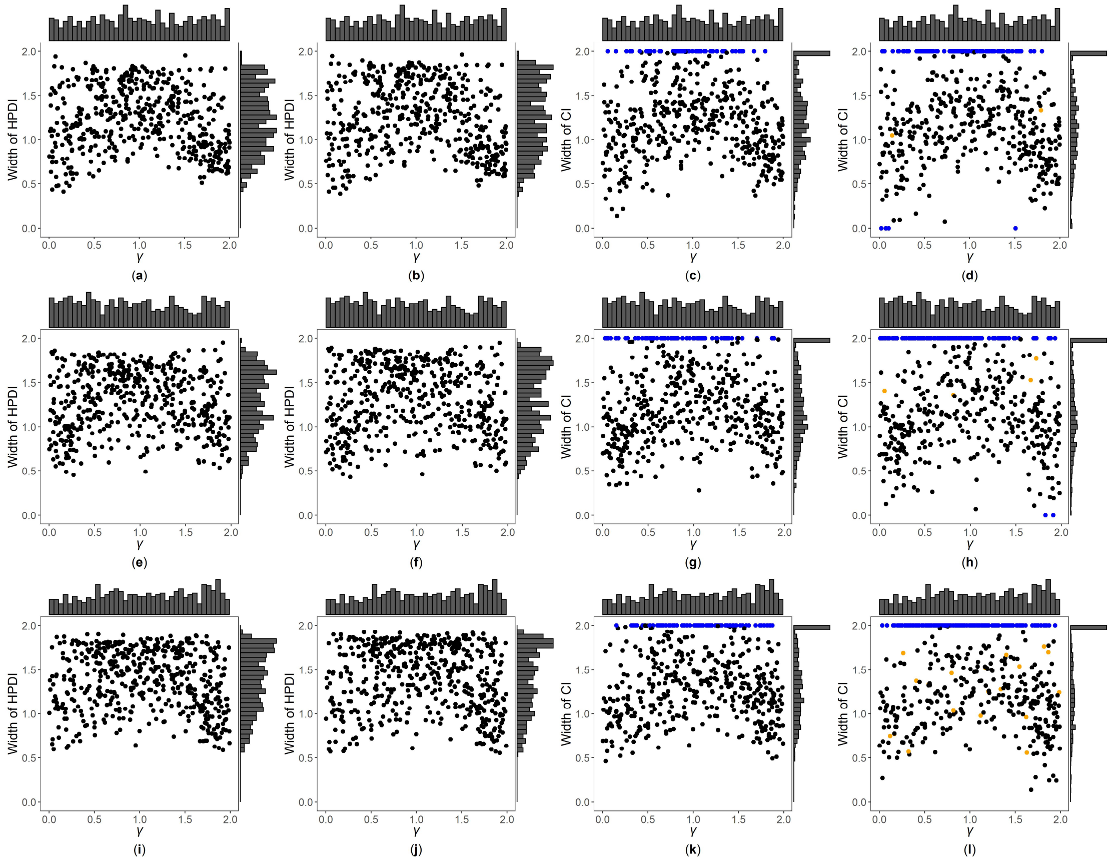

Figure S15. Widths of HPDIs or CIs of GBN, GBU, PF and Fieller's methods against true values of γ for quantitative trait with $n = 2000$, $\tau = 0.6$ and $\sigma = 2$. The blue points represent the widths of the empty sets or the noninformative intervals, and the orange points represent the widths of the discontinuous intervals. (**a**) GBN with $\eta = 0$; (**b**) GBU with $\eta = 0$; (**c**) PF with $\eta = 0$; (**d**) Fieller with $\eta = 0$; (**e**) GBN with $\eta = 0.4$; (**f**) GBU with $\eta = 0.4$; (**g**) PF with $\eta = 0.4$; (**h**) Fieller with $\eta = 0.4$; (**i**) GBN with $\eta = 1$; (**j**) GBU with $\eta = 1$; (**k**) PF with $\eta = 1$; (**l**) Fieller with $\eta = 1$.

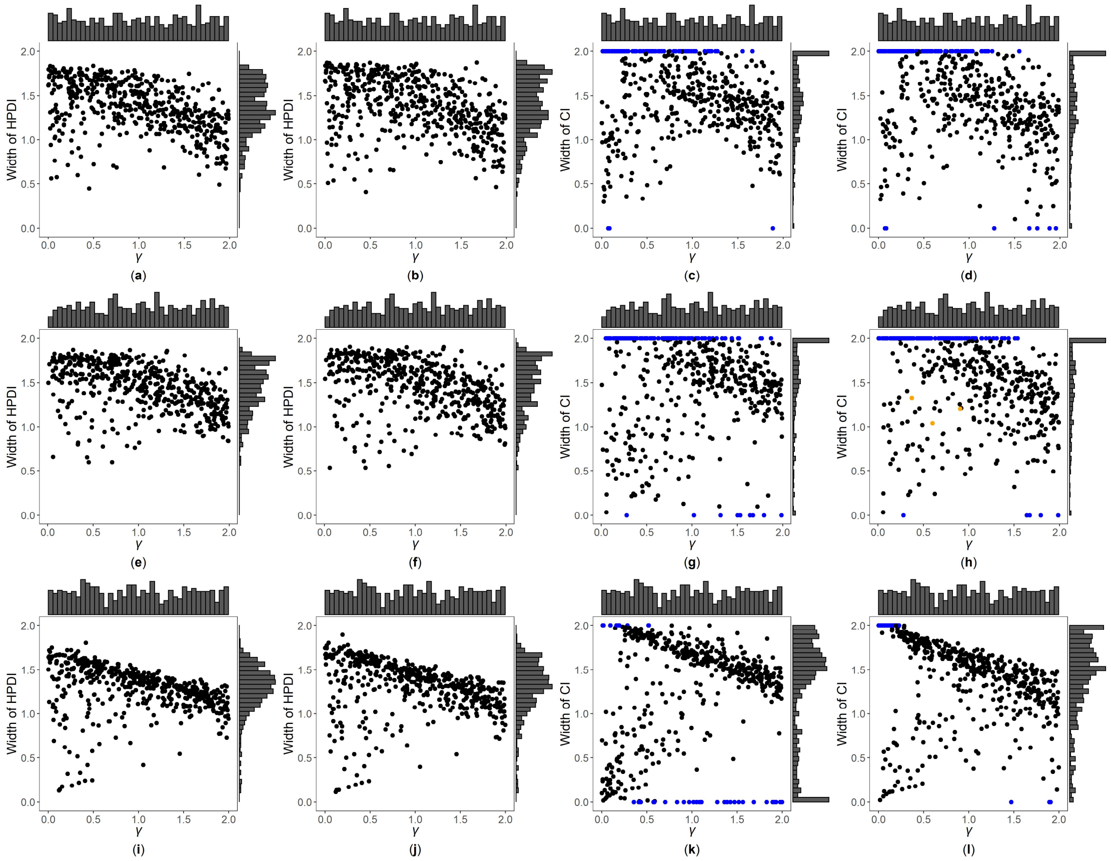

Figure S16. Widths of HPDIs or CIs of GBN, GBU, PF and Fieller's methods against true values of γ for quantitative trait with $n = 2000$, $\tau = 1$ and $\sigma = 2$. The blue points represent the widths of the empty sets or the noninformative intervals, and the orange points represent the widths of the discontinuous intervals. (**a**) GBN with $\eta = 0$; (**b**) GBU with $\eta = 0$; (**c**) PF with $\eta = 0$; (**d**) Fieller with $\eta = 0$; (**e**) GBN with $\eta = 0.4$; (**f**) GBU with $\eta = 0.4$; (**g**) PF with $\eta = 0.4$; (**h**) Fieller with $\eta = 0.4$; (**i**) GBN with $\eta = 1$; (**j**) GBU with $\eta = 1$; (**k**) PF with $\eta = 1$; (**l**) Fieller with $\eta = 1$.
